# A qualitative enquiry into participants’ and practitioners’ experiences in the Australian Liver FaIlurE trial

**DOI:** 10.1136/bmjopen-2024-089666

**Published:** 2025-02-16

**Authors:** Jeyamani Ramachandran, Anuradha Pati, Luisa Wigg, Sumudu K Narayana, Sharon Lawn, Kate Muller, Alan J Wigg

**Affiliations:** 1 Hepatology and Liver Transplantation Medicine Unit, Flinders Medical Centre, Bedford Park, South Australia, Australia; 2 College of Medicine and Public Health, Flinders University, Bedford Park, South Australia, Australia; 3 The University of Adelaide—North Terrace Campus, Adelaide, South Australia, Australia; 4 James Cook University—Townsville City Campus, Townsville, Queensland, Australia

**Keywords:** Clinical Trial, Health policy, Health Services

## Abstract

**Background:**

The Australian Liver FaIlurE (ALFIE) trial, a multicentre, randomised controlled trial, assessed the efficacy of a nurse-coordinated model of care to reduce liver-related emergency admissions (LREAs) in patients with decompensated cirrhosis. The model of care was delivered by a specialist nurse, including intensive postdischarge monitoring, linkage to multidisciplinary care, rapid access to care pathway, enhanced education and self-management support.

**Objective:**

To examine the experiences of participants and practitioners in the ALFIE trial to understand its impact, barriers and areas for improvement.

**Design and setting:**

A qualitative semistructured interview analysis nested within the ALFIE trial.

**Participants:**

A purposeful sample of 15 patients, 14 controls and 12 staff.

**Intervention:**

Thematic analysis of interview transcripts.

**Results:**

Interventional participants and the nurses perceived the care provided as personalised, holistic and continuous. The intervention enabled the development of robust therapeutic relationships and trust that promoted participant engagement and risk factor modification. It helped intervention participants navigate the busy hospital system. The control participants desired more education and a personal contact to deal with emergencies. With respect to the intervention, nurses felt that their support helped reduce LREAs and improve care, but it was overwhelming. A number of barriers and systemic issues were identified. Suggestions for improvement of the intervention model included increased staffing, improved mental health support and communication pathways with primary care practitioners.

**Conclusions:**

The ALFIE trial was well received by nurses and participants. It met the needs of intervention participants and the health system through easy-to-navigate, personalised, holistic and ongoing care. The study identified barriers and systemic improvement areas.

**Trial registration number:**

ACTRN12617001293358.

**STRENGTHS AND LIMITATIONS OF THIS STUDY**

- Semistructured interview design delivered by independent interviewers allowed free expression of participants’ perspectives and space to raise further insights.
- Purposeful sampling of control and intervention participants from all sites, thus minimising centre bias.
- Although blinding of interviews and coders was intended, due to the nature of the interviews, unblinding was unavoidable.
- The experiences of participants’ caregivers were not included in the study despite their vital role in patient care and support.
- An evidence-based implementation framework did not underpin the interview questionnaire.

## Introduction

 Cirrhosis of the liver results in multiple complications as it progresses from a compensated to a decompensated stage, including variceal bleeding, ascites and hepatic encephalopathy.^1^ It is often accompanied by recurrent liver-related emergency admissions (LREAs) associated with high mortality.^2^ Retrospective and single-centre small-scale studies have shown that careful monitoring of at-risk patients may be beneficial in preventing these hospitalisations.^3^,^6^ To provide the much-needed, high-quality evidence for a chronic disease management (CDM) intervention in decompensated cirrhosis (DC), the Australian Liver FaIlurE (ALFIE) trial, a multicentre randomised controlled trial (RCT) was conducted as detailed in supplementary file.^7^ The primary aim was to assess the efficacy of a nurse-coordination model of care (MOC) in reducing LREAs in DC. The care-coordination model did not demonstrate an overall reduction in the rate of LREAs or improved survival; however, a number of clinically relevant benefits were observed. These included a reduction in LREAs due to encephalopathy, a greater proportion of elective versus emergency admissions and several improved quality of care and patient-reported outcomes.

Since the intervention involved interactions between multiple stakeholders (participants and nurses; nurses and the health system; participants and the system), the strength and success of the intervention cannot be assessed by quantitative outcomes alone. It is important to know what participants and staff thought of the processes and examine patient-reported outcomes more deeply, before the intervention can be implemented widely. The barriers and challenges experienced must be understood so that the intervention can be improved accordingly. Qualitative methodology is best suited to capture the subjective nature of patients’ experiences.

The importance of qualitative studies alongside quantitative research in healthcare research is well recognised.^8^ Their role has evolved from mere feedback on the RCTs to codesign with a focus on consumer lived experiences. The distinctive contribution of qualitative research in not just interpreting study results but also in design and conduct is highlighted in a case study by Powell *et al*.^9^ Integrating a qualitative approach in the feasibility and main study of Screening for Atrial Fibrillation with ECG to Reduce stroke trial demonstrated the way for future RCTs. The role of qualitative methods right from screening, consenting and ongoing conduct of the trial led to improvements in study design, understanding the outcomes and highlighted crucial issues in implementation as well.^9^ One-on-one interviews with people—key stakeholders—involved is one of the best ways to find out how they perceive an intervention and its implementation.^10^ In a chronic complex disease such as DC, the impact of a CDM intervention is influenced by patient experiences, be it positive or negative. In a similar vein, feedback from medical care providers who are closely involved in the patient’s journey is vital to fine-tune the programme to ensure the trial meets its intended purpose. The aim of this study was therefore to understand the experiences of participants and medical staff involved in the ALFIE trial to obtain a holistic assessment of the intervention and its benefits and barriers. The study objectives were to analyse how the intervention participants perceived the intervention and to examine the views of nurses and doctors who delivered the intervention. We also sought the perspectives of the control participants on the standard care to compare and understand the effect of the intervention better.

## Methods

### Design

This research was guided by a qualitative descriptive framework. This approach was best suited to this research because the goal was to acquire perspectives of participants and experiences of medical staff in a natural setting of service delivery. A qualitative descriptive framework has the ability to show participants’ reality in everyday language and remain close to the data. The study results were analysed on the principles of systems theory as it examined the linear and cyclic interactions between patients and nurses (micro system), between nurses and doctors (interactions between disciplines and between team members delivering the MOC, meso system) and their interactions with the health system in hospitals and with general practitioners in the community (finally where MOC happens, macro system).^11^ Systems theory has been used to understand the interactions between healthcare professionals as it can detect patterns and identify potential reasons for failure of systems.^12 13^ The previous research by the authors on a nurse-led clinic model also informed the methodology by providing perspectives on purposeful sampling, broader issues involved in coordinated care and highlighting the layers involved. It also guided the interview questionnaire development and the thematic analysis.^14^ There was no involvement of public and patients in the study design.

### Setting and participants

The study examined the experiences of service users within the ALFIE trial (participants) and ALFIE service providers (doctors and nurses). The particulars of the trial are described in detail in the initial paper.^7^ Interview responses were obtained from control and intervention participants of ALFIE (patients being treated for cirrhosis of the liver and its various complications within hepatology units across Australia) and doctors and nurses working with those health services. The doctors in the study were gastroenterologists, all with a special interest in hepatology and with an average of 10 years of experience. Nurses were hepatitis nurses with up to 5 years’ experience, without any prior experience in the management of chronic liver failure complications or CDM programmes. They received training before the trial and continuous ongoing support during the trial.

### Recruitment

A purposeful sampling strategy as described by Palinka *et al* was used to capture the experiences of chosen control and intervention participants who completed the study from all participating sites.^15^ The initial recruitment plan was to recruit 32 consecutive participants (eight patients from each major site, with four patients from each study arm) over two 5-month periods: October 2020 to February 2021 (Block 1) and October 2021 to February 2022 (Block 2). The interview plan also included interviews with eight nurses and four gastroenterologists/hepatologists, with the four major hospital sites represented.

### Data collection

The semistructured interviews were conducted by two senior specialist nurses and a knowledgeable junior doctor training in gastroenterology using the interview guide as shown in online supplemental tables 1 and 2. The interview questions were designed to allow the interviewees to talk about their experiences with the intervention, their perspectives on system-level changes that could improve the intervention, and also potential challenges within the service system of care. The interviewers were not involved in the delivery of the trial intervention. The interviews were conducted by phone within 3 weeks of completion of the study, recorded and transcribed verbatim, then imported into Nvivo 14 for analysis.

### Data analysis

The authors’ prior research on nurse-led clinic model in cirrhosis informed the analytical methods used for this study. The analysis was performed by three authors (JR, AP and LW) using Braun and Clarke’s six-phase framework for thematic analysis, as listed below^16^ :

- Step 1: become familiar with the data.
- Step 2: generate initial codes.
- Step 3: search for themes.
- Step 4: review themes.
- Step 5: define themes.
- Step 6: write up.

AP and LW were senior medical students familiar with both patient and nurse interactions, as well as the principles of nurse-led cirrhosis care due to their involvement in similar prior research. They both shared the transcripts and generated initial codes. JR, as a senior author, reviewed all the transcripts and independently coded them (steps 1 and 2 of the Braun and Clarke process). The three researchers had a series of online meetings to review all the codes and generate the themes (steps 2 and 3). The emerging themes were discussed with AW, the principal ALFIE investigator, and SL, an experienced qualitative analyst, before the final themes were decided on (steps 4 and 5). This was followed by the last stage of writing up the manuscript (step 6). Research team members involved in the analysis were blinded to the interventional status of the patients until after the final themes were discussed. The research team maintained reflexivity during analysis and writing by keeping an electronic record of the discussions.

## Results

Twentynine participants (15 intervention and 14 controls, median age 57 years, 66% male) and 12 medical staff (eight nurses and four hepatologists) completed the interviews at the end of the follow-up period. 76% of the interviewed participants had alcohol-related cirrhosis with a mean (SD) Mayo Clinic End stage Liver Disease score of 16 (6). 15 participants (eight control and seven intervention) presented with ascites and three (two control and one intervention) had hepatic encephalopathy at initial admission. All the eight nurses were women and the three hepatologists were men. The interviews lasted from 20 to 40 min and were conducted within 3 weeks of study completion. Themes that emerged from these interviews reflected a range of enablers and barriers to the effective delivery, positive experience of the intervention, as perceived by participants and staff and needs for better care by the controls.

**Table 1:** Themes from intervention participants’ interviews

	Themes	Codes	Quotes
Enablers of improved care	Personalised and holistic care	Help with care navigation by approachable nurses	Pt ID 1010: If I have any problems or feel crook in any way or something’s changed in me, to give LN a ring straight away and they’ll recommend either go down, ring up the hospital, make an appointment to see my doctor or come in and see them sort of thing at any time.Pt ID 2002: I have fairly regular appointments and if there is something I’m concerned about, I know I can call LN and they’ll get on to the doctor or just advise me on their own.
		Clear and simplified presentation of medical updates	Pt ID 1010: They’re straightforward. They speak English to me, more or less, that I can understand. You know? They didn’t beat around the bush or anything else, straightforward sort of thing. The little questions that I ask, they respond to me without beating around the bush or anything else. They tell me the goods and the bads.
		Ongoing care and planned follow-ups	Pt ID 3030: The constant blood checks. You know, making sure that everything is okay. You know, that everything is okay.Pt ID 2002: I have fairly regular appointments.
		Derived psychological support	Pt ID 2018: I think, you know, as a psychological support. So, it wasn’t just the physical and medical support, but also the psychological support of knowing there was someone there.
	Improved understanding of disease	Education on diet, medications	Pt ID 1027: Education on what could happen, what’s going to happen.Pt ID 1024: What not to do and what to do type of thing. Yeah, you’re not supposed to eat certain things, you know what I mean, surely.
		Addressing risk factors	Pt ID 2002: Saying don’t drink any alcohol and don’t do the kilo of grapes every week and stuff.
		Regular medical update	Pt ID 1019: That was very positive because they could chase up some questions, if they didn’t know the answer to questions that I had, they could chase them up and get back to me a day or so later or whatever. So, they were very good.
	Improved disease management and self-management	Awareness of situations to seek medical help	Pt ID 4036: So, I know the signs to be aware of, if I have any black stools or black bleeding or anything like that, I go straight to Emergency because of the risk of varices or bleeding and obviously constipation, to avoid that, and maintain a good diet, abstinence from alcohol and making sure I’m not retaining fluid.
		Encouraged lifestyle modifications	Pt ID 1014: I’ve stopped drinking and also reduced my sugar intake as well as stayed away from salt and things like that.
		Motivation	Pt ID 1002: You guys are helping me, and I need to help myself. I do learn (self-manage complications of cirrhosis). I do learn how to control or how to improve it and I still in progress of doing it.
	Perceived sense of well-being	Confidence in care received	Pt ID 2018: Yeah. I mean, my prognosis, they didn’t think I’d survive at the time of my hospitalisation and you know, now my prognosis is really, really good as long as I keep doing what I’m doing. I have a very good chance of a long and healthy life.Pt ID 2002: I’m one hundred per cent better than when you would have last seen me.Interviewer: Great. That’s fantastic. Really good.Pt ID 2002: You know, I was on the verge of being put on the transplant list and I’m nowhere near that now.
		Felt supported	Pt ID 1027: (Felt supported) with my decisions I decided to make.
	Confidence in system and healthcare	More faith in medical professionals	Pt ID 2018: Um, just ongoing support and care and coordination between the hospital and my GP. And, knowing that if I was to deteriorate or if I was to have any issue, that I could always contact the Liver Clinic for support.
		Enthusiasm for clinical studies	Pt ID 2002: Like I said I’m very happy with the help I’ve been getting and I appreciate this because this is why people should go into studies more.
Barriers to improved care	Variable styles of care	Change in frequency of calls with personnel	Pt ID 1014: Well, the one I had before was marvellous. They would ring all the time. This one does not ring as much, but they are still a very lovely person.Pt ID 1011: But once they left it dropped off. So, I was happy, it was a great idea, but it did drop off.
	Burden of intervention	Paperwork	Pt ID 1018: Could start my own toilet paper factory.

The quotes are amended to avoid identifying the name or sex of the personnel involved.

LNliver nurse

### Themes from interventional participants’ interviews

### The value ofpersonalised,holistic and continuous care

The intervention participants reported a range of perspectives that described enablers and barriers to improved care. Within these two broad areas, themes from participant interviews, along with direct quotes to demonstrate each theme, are listed in more detail in table 1.

Participants appreciated the education provided on nutrition and medications. They valued regular updates on the disease status that were provided in a simplified manner. This in turn improved their understanding of the complex disease. They appreciated the personalised and holistic care provided by the nurses, stating that nurses were approachable, kept track of their progress and organised postdischarge follow-ups. They also derived emotional and psychological support from the nurses. It was also highlighted that an important advantage of the intervention was having a point of contact in the intervention nurse, rather than being lost in the medical system. They also received valuable insights regarding risk factors and how to keep them under control. They felt this contributed to better self-management and, overall, improved their sense of health and well-being. It also clarified the importance of their personal role in maintaining improved health status. The organised and unfragmented care offered by ALFIE trial left them feeling confident about the health system. They expressed enthusiasm to participate in further such studies to contribute to better health for all.

Study participation barriers involved completing surveys and questionnaires periodically, which was not well received by some participants. Change of nursing personnel made intervention participants aware of the differences in delivery style of the intervention. This was not exactly a disadvantage but was nevertheless reported.

### Control participants’ perspectives

### Disconnected care experiences

In stark contrast to intervention participants, control participants expressed the need for a more personalised education rather than just the provision of study materials. They highlighted the need for organised follow-ups and resented the need to seek emergency services when their health deteriorated. They desired more input on self-management and the coordinated care focus. Themes from control participants’ interviews, along with direct quotes to demonstrate each theme, are listed in table 2.

**Table 2:** Themes from control participants’ interviews

Themes	Codes	Quotes
Gaps in education	Limited or no education	Pt ID 3021: There was a pamphlet. I probably had more than one pamphlet surely.Pt ID 1037: Um, nothing more than I knew already and through my GP.Interviewer: Did you receive any education as regards to your liver disease?Pt ID 1015: No, not at all.
	Disease course and complications	Interviewer: What else did you learn about liver disease?Pt ID 2013: Not much.Interviewer: Did you learn about any of the complications of liver disease that might happen like what to watch for these complications?Pt ID 2013: No.
	Self-management plans	Interviewer: And, did you learn about self-management for the complications of liver disease?Pt ID 1009: No, not really. No. The doctors said to cut my drink out which I’ve done.
Lack of coordinated care	No personalised care	Pt ID 2003: Yeah, again it was more so, you know sort of me leading it. Yeah, I didn’t have sort of anyone call and say, hey, how are you going or anything like that.
	No follow-up plans	Pt ID 3023: Yeah, like I usually end up in hospital. I come into the Emergency Department.Interviewer: And, have you got any appointments coming up or any tests or anything you need to do for your liver that you know of?Pt ID 1009: No, I haven’t got anything.

The quotes are amended to avoid identifying the name or sex of the personnel involved.

### Themes from medical staff’s interviews

### Importance ofholistic care withincomplex and challenging systems

The medical staff also reported a range of perspectives that describe enablers and barriers to improved care. Themes from medical staff interviews reflecting the enablers of improved care and barriers, along with direct quotes to demonstrate each theme, are listed in more detail in tables3  4, respectively.

**Table 3:** Enablers of care from staff interviews

Themes	Codes	Quotes
Holistic care	Clinical, emotional and psychosocial support	Staff 4: Yeah, reminders like, how are you going? Do you need some more, you know, another ascitic tap rather than just letting them go on and getting sicker and sicker.
	Nutritional support	Staff 1: Through the conduit of the therapeutic relationship, we were able to repeatedly come back to fundamental issues regarding nutrition.
	Address risk factors such as alcohol	Staff 5: I think it’s a really relevant role and I think it does a lot of good, you know, there were three patients that I know with heavy substance abuse who claim outright that having that nursing role there is what saved their lives and stopped them from drinking.
	Assistance with non-medical community supports	Staff 5: So, it really is a lot more of the social, I kind of became a case manager for these patients who wanted engagement.
Personalised care	A point of contact accessible at times of need	Staff 10: Having a person, a go to source, someone to call if they are noticing more ascites or something’s a bit different. I think that was one of the benefits for the patients.Staff 12: We are a main point of contact.
	Customised medical care	Staff 8: … develop a patient centred care plan for them and also it is convenient for the patient that they know that if something happened, that they know who to contact, where to get the help.
	Titration of diuretics, management of hepatic encephalopathy	Staff 7: Diuretic management was one and titrating the management of encephalopathy, those two were predominantly the most challenging for them.
	Development of trust and therapeutic relationship	Staff 8: I think with the case management we know the patient more and also, we have a very good relationship with the patient which enables the patient to tell us more about their problems.
Continuous care	Follow-up phone calls facilitating patient engagement	Staff 5: Um, I think the phone calls, you know, the first weekly phone calls in the first 12 weeks really helped 2/2 develop a good relationship, a good relationship with the patient and their nurse and from then I think that set up for then to be able to develop that trust and turn to them if they needed. If you have an engaged patient.
	Follow-up during hospital admissions	Staff 5: Going through their medications constantly reviewing their medications every time they had hospital admissions.
	Postdischarge follow-ups	Staff 4: Having someone monitoring their bloods when they had their diuretics titrated, so they didn’t have to do three trips back to the doctor’s or chase around phone calls.
Care navigation	Liaison between patients and healthcare system	Staff 12: And, I think having a nurse in between of all that helps because they can just call us and we are able to help liaise between say, the specialist, or discuss with another doctor or encourage them to go to their GP and tell the GP like, hey, this is probably what you should do. I just think it helps also in escalating care a lot faster.Staff 3: I think one of the things is that they ring the hospital, they used to get the run around and it’s given them a port of call and a place where they can get information and hopefully, I can act on the information and get things done.
Reduce burden on doctors	Reduce hospitalisation	Staff 5: Yep. I feel that it (education) will up-skill or increase their knowledge and they will be more compliant and that ultimately reduce their admissions.Staff 7: Strengths was obviously having a nurse practitioner who could provide almost continuous management of the patient between clinic times where they were able to follow through patients’ issues of decompensated liver disease and to a point of hopefully minimising hospitalisation. I think there is a critical need for a role like that which can’t be provided by doctors.
	Reduce clinic appointments	Staff 6: Having someone monitoring their bloods when they had their diuretics titrated, so they didn’t have to do three trips back to the doctor’s or chase around phone calls, you know, it made life easier and took away a lot of the fatigue they get from the burden of appointments.
Education	Ongoing education on medications	Staff 2: Definitely medication management. Some patients have no idea what medications they’re on or they don’t understand the importance of being on them and having to take them every day.
	Compliance with surveillance	Staff 2: Again, explaining to the patient the need for ongoing surveillance.
	Encouragement and support for clinic attendance	Staff 5: A lot of patients think that the appointments aren’t important enough … so, just that encouragement they need to actually come to their appointments.Staff 2: Just being able to provide the ongoing education and support for them. I think it has just allowed them to have better rapport and with the doctors.
Professional development	Evolved confidence	Staff 7: I think it (confidence)was an evolving thing. There were times where I didn’t feel confident, but on those occasions, I was part of a multidisciplinary team and so I contacted the specialist, the relevant specialist on those occasions. I also contacted the LN who trained on a few occasions as well just for a bit more guidance. I regularly went back to the information that they provided in terms of specific aspects of case management.
	Improved knowledge and disease management	Staff 1: Yeah, and I would say that my skills in case management grew exponentially as a result of being involved in the ALFIE study.Staff 5: Um, yeah, I had to up-skill pretty quickly in medication titration related to paracentesis and ascites management.
	Understanding lifestyle factors in cirrhrosis of liver	Staff 1: Alcohol addiction and how it’s like this cycle, cycles within cycles. It’s not a linear thing where you get to the end of the problem and you know, you kind of move on. It’s, you’ve really got to hold on for the journey because for most people it’s a cyclical thing and it has wins and there’s lots of losses, so it’s a struggle.
	Improved communication and development of a therapeutic relationship	Staff 1: But this is the first time that I’ve really been able to work intensively on a practical level and on an individual level to being able to find out how we can find solutions, how we can form a therapeutic relationship with a patient that’s effective on those level.Staff 4: So, I reckon I got out of it the fact that for me, I developed a lot of nice communication and good relationships with patients.

The quotes are amended to avoid identifying the name or sex of the personnel involved.

LNliver nurse

**Table 4:** Barriers to care as expressed by staff

Themes	Codes	Quotes
Lack of resources	Lack of mental health resources	Staff 5: I think we had limited resources in our hospital for situations like that, you know, a lot of the time we had to redirect the patients through Emergency and it would have been better to be able to give them, you know, some sort of drug and alcohol counselling or get them into a detox unit if they wanted to or something in the community, but we just didn’t have those resources.Staff 1: For example, when trying to refer two patients to an addiction psychiatrist, that was also difficult to eventually find someone.
	Nurses’ inability to cater to mental health needs	Staff 1: Felt under-skilled in the psychosocial emotional area because the patients kind of look to you, you know … in terms of alcohol addiction.Staff 1: Think that’s just an enormously challenging area and I think as, you know, I felt that I was getting somewhere with some patients and then they’d fall off the wagon and my confidence in whether I did the right thing, whether I said the right things, did decline on those occasions and it didn’t recover very quickly.
	Coordinating all of patients’ needs	Staff 3: Think the weaknesses of the intervention was I think was that it hasn’t quite got it’s referral. We only sort of started the infusion clinic this year or last year, so it’s a new thing and the skill and like, for instance, one of our patients, he used to have 10 litres in his ascites and tyring to coordinate an ultrasound so that they could do an ultrasonic tap on him, we had to send him to Westmead, so it was not quite getting the radiology on board to support some of the services.
Time consuming	Additional paperwork	Staff 8: The weakness, it would be, to be honest, very time-consuming and it takes a lot of time and also to coordinate the different surveys and another thing is to trace all the paperwork.
	Inadequate staffing	Staff 12: I find staffing is the really big weakness because unfortunately on our site I’m the only person doing the study and I obviously can’t put one hundred per cent into it because of the fact that it is only one person.
Overwhelming	The level of patient sickness	Staff 1: … and I found that I didn’t have the support that I needed personally to be able to deal with some of the disappointments and then, I guess, seeing your patients decline rapidly before your very eyes is very upsetting.
	With patient numbers	Staff 1: So when you are involved in a large number of patients, they will be overwhelmed.
Complexity of care	Complicated medical presentations	Staff 4: They were too sick for me to look after and manage on an outreach basis, but I constantly had to say to them, ‘well, please come to the hospital’. Their needs were greater, either they were anaemic or they needed a tap or they were significantly short of breath or they were having some other issue that needed to be resolved medically.
	Sick patients	Staff 1: Needs of the patient which diverts hugely from the expectations set by the visit schedule, so I ended up devoting a lot more time given the complexity and the level of sickness of the patients, yeah, a lot more time than I was kind of expecting.
	Need for experience and a good working knowledge of liver diseases	Staff 5: I think you definitely have to have a good understanding and knowledge of liver, you know, liver problems and you definitely need to be a specialist in liver as well as also you need to have a degree of competency in counselling and sort of providing emotional advice.
Safety concerns	Angry patients	Staff 6: I had one in particular who would just ring and swear at me.
	Unhappy patients	Staff 9: Well, because some patients, they didn’t like being called quite frequently.
Conditional benefit	Patient engagement and motivation	Staff 1: The patients that it seemed to help the most were the patients who really wanted to engage with the way.Staff 5: Only if you had an engaged patient Like I said, you know, it really works if the patient’s motivated and it would be good to see it expanded to a bigger group.Staff 6: Like I said, you know, it really works if the patient’s motivated.Staff 9: We have a cohort of patients who don’t want to do anything irrespective of what you do, we can’t change those patients.

The quotes are amended to avoid identifying the name or sex of the personnel involved.

LN, liver nurse

The staff were happy with the holistic, personalised care that they provided through ALFIE. The nurses strongly felt that the ongoing continuous care in ALFIE addressed key complications of cirrhosis (namely, ascites and hepatic encephalopathy) and prevented patients from presenting too late. They felt that their advice on nutrition and risk factors addressed disease progression. Nurses were happy to coordinate various community supports, thus providing a truly holistic care. They felt that their easy accessibility and service as a point of contact enabled patients to reach out to them during times of deterioration. They felt that their regular communication with participants encouraged hospital attendance and the need for regular surveillance procedures. Importantly, they felt that their ongoing customised care prevented emergency presentations and helped patients avoid and economise clinic visits. They also viewed the trial experience as a professional development opportunity. Their understanding of liver disease improved, as well as their confidence in navigating a complex chronic disease. They enjoyed regular communication at a personal level with the participants, and development of this robust therapeutic relationship ensured ongoing participant engagement.

The major barrier reported by the staff was inadequate staffing. The care of patients with DC was perceived as overwhelming and time consuming. The major contributing factors identified were complexity of the participants’ clinical condition and the multiple medical needs that needed to be addressed within time constraints. The additional paperwork for the trial complicated the time factor. Their overall impression was that the care was demanding due to the high level of morbidity and need for frequent hospitalisations. Frequent encounters with mortality and intensive care admissions left the staff demoralised. They felt that the benefits provided by ALFIE were conditional in that, predominantly, engaged and motivated participants derived the most help and benefit from the intervention.

### Key challenges and suggestions for improvement

**Figure 1:**
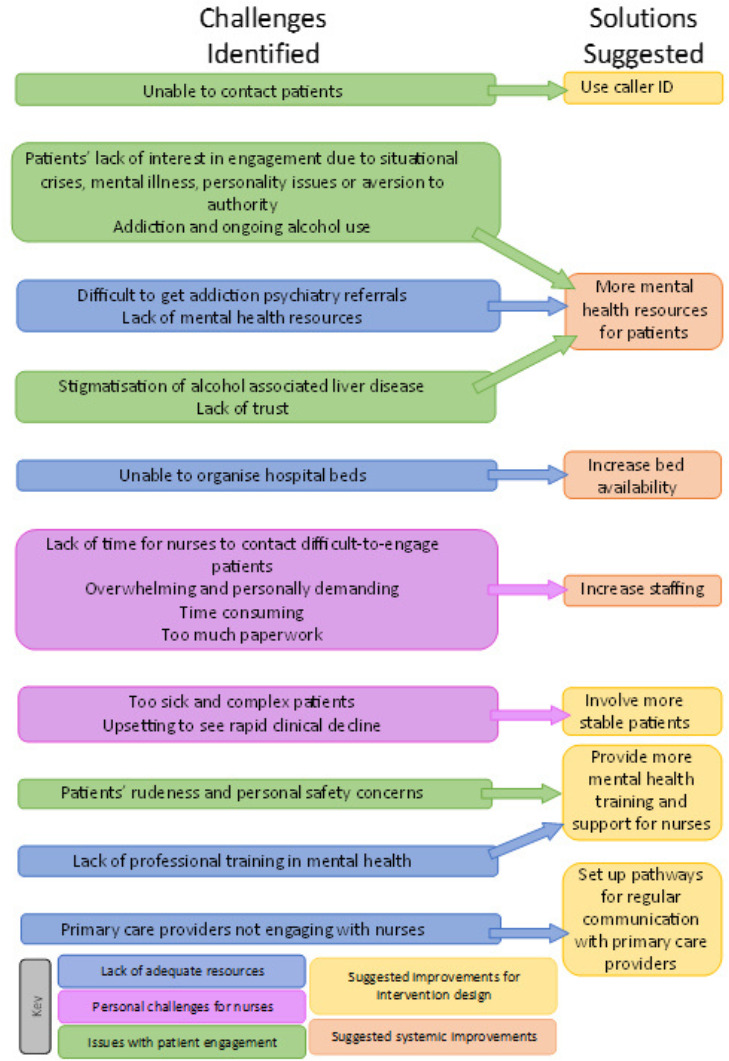
Challenges faced by the staff and suggested solutions to improve the intervention.

Improving staffing to a full-time position rather than the part-time (3 days per week) resource used in the study was a crucial suggestion. The nurses also felt ill-equipped to provide the mental health support that was often required. In addition, having to contact patients with unstable living situations was a big undertaking. The staff struggled with patients who were unwilling to engage with the intervention and were impolite. Having to organise a hospital bed for sick patients with worsening health was also challenging, often due to the limited availability of beds. When staff attempted to involve primary care providers in patient care, it was not universally welcomed by all primary care providers. Suggestions to address the challenges experienced are listed in figure 1. The nurses also expressed interest in looking after stable chronic patients as well as patients with DC, which consumes a lot of time and resources.

### Interlinked benefits

**Figure 2:**
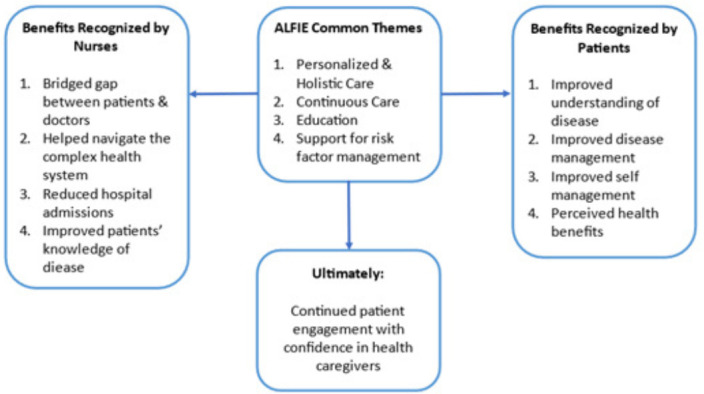
Mind map illustrating the overlap between participants’ and staff’s observations on Australian Liver FaIlurE (ALFIE).

The intervention was perceived by both patients and staff as providing continuous, personalised and holistic care, thus supporting patients in multiple health and well-being domains, including psychosocial support, symptom management, nutrition and risk factor management. Staff felt that the intervention bridged the gap between participants and doctors. Additionally, intervention participants appreciated having accessible care that helped them navigate a complex system. Ultimately, the intervention resulted in better self-management and disease management with perceived health benefits and improved patient confidence. This overlapping interaction is illustrated in figure 2.

## Discussion

The study investigated the experiences of both participants and the staff who provided care in the ALFIE study. Although the intervention was not successful in achieving reduced emergency admissions, it did reduce admissions due to hepatic encephalopathy and improved quality of life.^17^ Given the emerging importance of patient-reported outcomes in clinical studies, the current study findings are relevant.

In addition to experiencing the benefits from advice on nutrition, medication management and management of risk factors for liver cirrhosis, participants in the intervention arm appreciated the provision of personalised care in the ALFIE intervention. This highlighted the important role of the nurses in the care navigation process, not just the performance of tasks. Having a point of contact in the medical system helped intervention participants access medical assistance during emergencies and get most out of clinic visits. On the other hand, organised follow-ups were not available to control participants whose only option at times of need was to present to emergency without any care navigation or support. A 2004 literature highlighted poor understanding of the health system to be an impediment to patients accessing appropriate care.^18^

The importance of holistic care that accounts for the physical and emotional well-being of the patient is highlighted in recent publications as an important mechanism in coping with chronic conditions and their management.^19^ The intervention promoted a sense of well-being beyond medical management by means of positive interactions. In DC, where cure is not often possible, ongoing patient engagement is essential for symptom management with dietary restrictions and compliance with medications, risk factor reduction and surveillance (both radiological and endoscopic). The therapeutic relationship that emerged between intervention participants and ALFIE nurses enhanced their engagement and confidence in the health system.

Intervention participants reported that improved disease understanding encouraged them to make the right choices. Their expression of being motivated, feeling confident about care received and improved understanding of disease reflect the core principles of self-management. Patient activation is a critical concept that underpins the success of chronic care models.^18^ In the example of diabetes, patient participation was shown to contribute to better glycaemic control and quality of life.^20^ It seems likely that this factor is often overlooked in the management of a complex chronic disease such as DC. It is interesting that the participants’ perception of improved self-management was supported by higher scores in the Partners in Health scale (validated measure of self-management in cirrhosis) in the primary study.^21^ In comparison to the control group, significant improvements were noted for the intervention group in the partnership and symptoms domains of the Partners in Health scale.^7^

The perceived need by the control participants for personalised advice on nutrition and disease management showcased the inadequacy of presentation of information solely by way of printed material. These multimorbid chronic patients are better supported if the provided information is also applied to their situation by means of dedicated education sessions. This was provided to intervention participants and reported as missing by the controls. This point supports the need for personalised intervention, such as ALFIE, in cirrhosis management.

A large international study conducted among nurses across various European hospitals reported the following neglected areas in nurse-patient interactions: ‘comfort/talk with patients’ (53%), ‘developing or updating nursing care plans/care pathways’ (42%) and ‘educating patients and families’ (41%).^22^ It is not surprising that the nurses in the ALFIE study appreciated the opportunity to connect with patients and offer aspects of care that are often neglected in routine care such as patient education, nutritional advice and psychological support.

Evidence in favour of nurse clinics catering to the needs of patients with chronic disease is mounting.^23 24^ In the ALFIE study, person-centric care was provided by nurses who had more time than doctors in meeting patients’ needs for tailored information and coordinated care with regular postdischarge follow-ups. In a prior study, our group also reported patients’ and nurses’ positive perceptions of the care delivered in nurse-led community clinics for stable cirrhosis.^14^ The ALFIE nurses were keen to manage stable compensated cirrhosis as a change from seeing only sicker decompensated patients. Integrating management of stable cirrhosis in the existing chronic disease clinics in the community is likely to be an important future goal for large liver centres given the increasing prevalence of cirrhosis and the mismatch in availability of specialist appointments.

One of the key challenges faced by the nurses was limited access to mental health referral pathways and addiction specialists. The study also identified knowledge gaps and lack of confidence and training of hepatology nurses in mental health. It is not uncommon for severe mental illness and liver disease to coexist.^25 26^ A recent review has highlighted a high frequency of mental health diagnoses in patients with alcohol misuse and liver disease.^27^ It seems highly desirable therefore that nurses who provide a continuous care for cirrhosis in CDM models be equipped with skills to provide mental health support. Lack of mental health training for nurses in primary healthcare was demonstrated in an integrative review of 13 publications by McInnes *et al*.^28^ The study revealed poor knowledge, lack of training and confidence among primary health nurses. Interestingly, positive outcomes of training programmes were also reported ranging from increased confidence and better referrals.^28^ In line with the recommendations of the Second World Congress on Integrated Care in Sydney, integrating physical and mental health will benefit patients with cirrhosis by addressing risk factors and promoting engagement with services. One of the key findings from this study was therefore the need to strengthen the CDM model by both upskilling of hepatology nurses and improved linkages with a multidisciplinary team including psychologists and addiction specialists to provide service on demand.^29^

A few limitations of our study need to be acknowledged as well. The study did not report on the experience of caregivers. Caring for patients with DC is overwhelming due to the multiple hospital visits, frailty, symptom burden and loss of wages,^30^ and their perspectives would have provided further valuable information about the usefulness of our intervention. The interviewers and coders were blinded to the interventional status of the participants. However, during the process of interviewing and coding, unblinding happened unintentionally. Additionally, a pre- and post-ALFIE interview may have also provided a richer perspective on the experiences of both control and intervention participants.

Despite these limitations, a strength of the study is that it is the first to provide detailed qualitative feedback from patients with DC interacting with and without a CDM model. Additionally, a rigorous methodology was used to capture the lived experiences of both controls and intervention participants, nurses and doctors. These experiences were recorded until thematic saturation was achieved and were coded by three independent researchers blinded to the interventional status, then reviewed by a senior qualitative analyst.

## Conclusions

ALFIE was well received by participants and staff alike. It enabled continuous unfragmented care that was proactive and responded to many of the patients’ needs. The intervention delivered by nurses served as a core bridge between patients and doctors and filled the gaps in the system that were often overlooked. It helped build patients’ capacity to deal with changing disease course, improve understanding, self-manage and support their decision-making. The intervention empowered nurses with new knowledge about complex disease management. However, care of patients with DC placed a huge demand on the nurses’ time, resources and mental well-being. The study identified important modifiable barriers that could be overcome to improve the intervention in potential future studies. An important barrier was inadequate staffing. To improve care and prevent nurse burnout, future models should attempt to appropriately match nursing resource to patient load, rather than the part-time (3 days per week) resource used in our study. Augmentation of the model with more accessible mental health referral pathways and enhanced engagement with primary care providers are other barriers that need to be addressed before wider implementation. Importantly, the study highlighted the lack of support to control participants in education and organised follow-ups, emphasising the need for more personalised and coordinated CDM in cirrhosis.

## supplementary material

10.1136/bmjopen-2024-089666 online supplemental file 1

10.1136/bmjopen-2024-089666 online supplemental table 1 and 2

## Data Availability

Data are available upon reasonable request.
