# Supplementary material for: A qualitative enquiry into participants’ and practitioners’ experiences in the Australian Liver FaIlurE trial
Source: BMJ Open. 2025 Feb 16;15(2):e089666. doi: 10.1136/bmjopen-2024-089666 (PMC11831275; doi:10.1136/bmjopen-2024-089666)
Supplement: online supplemental file 1 [file bmjopen-15-2-s001.docx]

**Efficacy and cost effectiveness of a chronic disease management model**

**in patients with decompensated cirrhosis.**

**The Australian Liver Failure (ALFIE) trial.**

Sponsor

Flinders Medical Centre

Hepatology and Transplant Medicine Unit

c/- Level 3 Gastroenterology

Flinders Drive

Bedford Park SA 5042

TEL: 08 8204 6989

FAX: 08 8204 3943

18 September 2020

CONFIDENTIAL

The information contained herein is the property of the Hepatology Department at FMC

and may not be reproduced, published, or disclosed to others without the

written authorisation of Assoc. Prof Alan Wigg.

Statement of Compliance

This study will be conducted in compliance with all stipulation of this protocol, the conditions of the ethics approval, the NHMRC National Statement on Ethical Conduct in Human Research (2007) and the Note for Guidance on Good Clinical Practice (CPMP/ICH-E6).

Contents

[Glossary of Abbreviations and Terms 4](#_Toc22127203)

[Study locations and project team 5](#_Toc22127204)

[Project Team 5](#_Toc22127205)

[Sir Charles Gairdner Hospital 5](#_Toc22127206)

[The Queen Elizabeth Hospital 5](#_Toc22127207)

[Lyell McEwin Hospital 5](#_Toc22127208)

[Royal Adelaide Hospital 5](#_Toc22127209)

[Blacktown Hospital 5](#_Toc22127210)

[Planned Timeline 5](#_Toc22127211)

[Background 6](#_Toc22127212)

[Study Objectives 10](#_Toc22127213)

[Primary Objective 10](#_Toc22127214)

[Secondary Objectives 10](#_Toc22127215)

[Research Plan and Methods 11](#_Toc22127216)

[Trial design 11](#_Toc22127217)

[Study duration 11](#_Toc22127218)

[Number of Participants 11](#_Toc22127219)

[Treatment Assignment 11](#_Toc22127220)

[Participant Population 11](#_Toc22127221)

[Participant Inclusion Criteria 11](#_Toc22127222)

[Participant Exclusion Criteria 11](#_Toc22127223)

[Consent of potential participants 11](#_Toc22127224)

[Intervention and 13](#_Toc22127225)

[The Chronic Disease Management Model: 13](#_Toc22127226)

[Role of CLD nurse 14](#_Toc22127227)

[Role of Gastroenterologist/Hepatologist 14](#_Toc22127228)

[Role of General Practitioner 14](#_Toc22127229)

[Control/usual care group 14](#_Toc22127230)

[Adverse Events 15](#_Toc22127231)

[Treatment of Participants 15](#_Toc22127232)

[Concomitant medications 15](#_Toc22127233)

[Randomisation 15](#_Toc22127234)

[Assignment of Site and Participant Numbers 15](#_Toc22127235)

[Visit Procedures 15](#_Toc22127236)

[Pre-Screening 15](#_Toc22127237)

[Baseline 15](#_Toc22127238)

[Baseline – all patients 16](#_Toc22127239)

[Baseline – intervention only 16](#_Toc22127240)

[Home Visit 16](#_Toc22127241)

[Telephone Contact 17](#_Toc22127242)

[Outpatient review by Specialist Gastroenterologist/Hepatologist 17](#_Toc22127243)

[Month 3, Month 6, Month 12, Month 18 17](#_Toc22127244)

[End of Study 17](#_Toc22127245)

[Outcomes 18](#_Toc22127246)

[Unplanned liver-related hospital admission rate 20](#_Toc22127247)

[Incremental cost effectiveness relative to usual care and quality of life 20](#_Toc22127248)

[Mortality and disease severity 20](#_Toc22127249)

[Quality of care indicators 20](#_Toc22127250)

[Patient Satisfaction and Self-Management Ability 20](#_Toc22127251)

[Intervention period 21](#_Toc22127252)

[Statistical Methods 22](#_Toc22127253)

[Sample size and power analysis 22](#_Toc22127254)

[Randomization, stratification, blinding 22](#_Toc22127255)

[Statistical methods 22](#_Toc22127256)

[Trial Management 23](#_Toc22127257)

[Training 23](#_Toc22127258)

[Data Monitoring 23](#_Toc22127259)

[Schedule for CONTROL patients 24](file:///\\fusafmcf01\Surgery\S&SS\Clinical%20Unit\Gastroenterology\Gastro\Chronic%20Liver%20Disease\CLINICAL%20RESEARCH%20TRIALS\ALFIE\Protocol\CURRENT\Australian%20Liver%20Failure%20(ALFIE)%20Protocol%20V10%2003SEPT2019_clean.docx#_Toc22127260)

[Schedule for INTERVENTION patients 25](file:///\\fusafmcf01\Surgery\S&SS\Clinical%20Unit\Gastroenterology\Gastro\Chronic%20Liver%20Disease\CLINICAL%20RESEARCH%20TRIALS\ALFIE\Protocol\CURRENT\Australian%20Liver%20Failure%20(ALFIE)%20Protocol%20V10%2003SEPT2019_clean.docx#_Toc22127261)

[References 27](#_Toc22127262)

[Appendices/Data Collection Tools 30](#_Toc22127263)

[Appendix A – Baseline Collection Tool 30](#_Toc22127264)

[Appendix B – Patient Identification Card (intervention only) 32](#_Toc22127265)

[Appendix C – Ascites and Hepatic Encephalopathy Assessment 33](#_Toc22127266)

[Appendix D – Osteoporosis Assessment 34](#_Toc22127267)

[Appendix E – Alcohol Assessment 35](#_Toc22127268)

[Appendix F – Variceal Management Assessment 36](#_Toc22127269)

[Appendix G – Nutrition Assessment 37](#_Toc22127270)

[Appendix H – Carer and GP Assessment 38](#_Toc22127271)

[Appendix I – Home visit form 39](#_Toc22127272)

[Appendix J – Hospital Admission Log 41](#_Toc22127273)

[Appendix K – Outpatient Attendance Log 43](#_Toc22127274)

[Appendix L – West Haven Criteria 45](#_Toc22127275)

[Appendix M – Contact Log 46](#_Toc22127276)

[Appendix N– Child Pugh and MELD Score 47](#_Toc22127277)

[Appendix O – Co-morbidities 48](#_Toc22127278)

[Appendix P – Concomitant Medications 49](#_Toc22127279)

[Appendix Q – End of Study Assessment Form 50](#_Toc22127280)

[Appendix R – HCC Assessment form 52](#_Toc22127281)

[Appendix S – Care Plan 53](#_Toc22127282)

[Appendix T – Charlson Comorbidity index 55](#_Toc22127283)

[Appendix U – Diuretic Titration Guidelines for Trial Nurses 58](#_Toc22127284)

# Glossary of Abbreviations and Terms

CDM – Chronic Disease Management

CLD – Chronic Liver Disease

CLDQ – Chronic Liver Disease Questionnaire

CM – Case Management

FMC – Flinders Medical Centre

GP – General Practitioner

HCC – Hepatocellular Carcinoma

HCV – Hepatitis C Virus

HE – Hepatic Encephalopathy

LMH – Lyell McEwin Hospital

NAFLD – Non-alcoholic fatty liver disease

PIH – Partners In Health

QEH – Queen Elizabeth Hospital

RAH – Royal Adelaide Hospital

RCT – Randomised Controlled Trial

SBP – Spontaneous Bacterial Peritonitis

SCGH – Sir Charles Gairdner Hospital

# Study locations and project team

## Project Team

The project team will include the following investigators:

- A/Professor Alan Wigg; hepatologist FMC, chief investigator
- Jeyamani Ramachandran; hepatologist FMC, Flinders University PhD student
- Kate Muller; hepatologist FMC
- Professor Richard Woodman; biostatistician FUSA
- Billingsley Kaambwa; health economist FUSA
- Rachel Wundke – nurse educator, CLD Nurse FMC, Trial Coordinator
- Rosemary McCormick- nurse educator, CLD Nurse FMC, Assistant Trial Coordinator
- Libby Bambacas – Hepatology Department Clinical Trial Manager
- Sumudu Narayana – Hepatology Department Clinical Research Assistant

### Sir Charles Gairdner Hospital

- Leon Adams; hepatologist SCGH, principal site investigator SCGH
- Garry Jeffrey; hepatologist SCGH, Sub investigator
- SCGH CLD nurse

### The Queen Elizabeth Hospital

- Dep Huynh; hepatologist QEH, principal site investigator QEH
- Gary Nind- hepatologist QEH, Sub investigator
- Marcus Teo; hepatologist QEH, Sub investigator
- QEH CLD nurse

### Lyell McEwin Hospital

- Asif Chinnaratha; hepatologist LMH, principal site investigator LMH
- Damien Harding; hepatologist LMH, Sub investigator
- LMH CLD nurse

### Royal Adelaide Hospital

- Edmund Tse; Hepatologist RAH, principal site investigator RAH
- John Bate; Hepatologist RAH, Sub investigator
- RAH CLD nurse

### Blacktown Hospital

- Golo Ahlenstiel, Hepatologist Blacktown Hospital, principal site Blacktown Hospital
- Blacktown Hospital CLD nurse

# Planned Timeline

April 2020

April 2022

September 2018

First patient randomised

Last patient randomised

EOT

Intervention starts

Intervention ends

Each patient will receive 24 months of intervention will be different for each patient.

# Background

**Cirrhosis and chronic liver failure**

A variety of insults to the liver can lead to progressive fibrosis and cirrhosis. The most common causes in Australia include; alcohol abuse, chronic hepatitis C (HCV) infection, and non-alcoholic fatty liver disease (NAFLD). Ongoing injury to a cirrhotic liver may result in decompensation of liver functions or chronic liver failure (CLD). CLD is manifested by complications such as; ascites, variceal bleeding, encephalopathy, sepsis (most commonly spontaneous bacterial peritonitis) and renal dysfunction, protein calorie malnutrition and hepatocellular carcinoma (HCC). The prognosis following the onset of CLD is poor. Patients with early CLD (Child Pugh class B) have a 2-year survival of only 60% and this falls to 35% in advanced CLD (Child Pugh class C)([1](#_ENREF_1)).

**Prevalence and economic impacts of CLD in Australia**

Cirrhosis and CLD have a high prevalence and significant economic impacts in Australia. In Australia it is estimated that 6.17 million people are affected by liver diseases which result in 7,266 deaths in 2012 ([2](#_ENREF_2)). The direct health expenditure (hospital admissions, out of hospital medical services, prescription pharmaceuticals) associated with liver disease was estimated at $386.2 million per annum in 2012. The total financial cost of liver disease in 2012 including direct health costs, productivity impacts, informal carer costs, loss of taxation revenues and disability adjusted life years has been estimated at $50.7 billion dollars ([2](#_ENREF_2)). These high costs reflect the significant disability, morbidity and mortality associated with CLD. These costs are 40% more than those associated with type 2 diabetes and represent 39% of the annual cost of cardiovascular disease, one of the most costly conditions in Australia.

These costs are likely to increase substantially in the future reflecting a steady rise in the prevalence of CLD in Australia. Evidence for this increase comes from a variety of sources. Firstly, deaths from cirrhosis and CLD in Australia have increased 20% between the years 1999 and 2008 ([3](#_ENREF_3)). An analysis of ICD-10 codes for CLD diagnoses shows a national increase of 25% in hospital admissions for CLD between 2004/5 to 2007/8 ([4](#_ENREF_4)). The reasons for this increase are likely to be associated with the growing epidemics of alcohol abuse, hepatitis C and obesity within Australian communities. Three available National Health surveys indicate that the proportion of Australians drinking at hazardous levels has increased from 8.2% to 13.4% ([5](#_ENREF_5)). Increased per capita alcohol consumption ([6](#_ENREF_6)) and increased hospital admissions for alcoholic liver disease ([7](#_ENREF_7)) provide further evidence of increasing patterns of alcohol use in Australia. In addition, despite the arrival of highly effective antiviral therapy for HCV it is anticipated that without substantial increases in treatment uptake a three-fold increase in numbers of patients cirrhosis, HCC and liver-related death by 2030 ([8](#_ENREF_8)). The rising rates of obesity (from 38% of Australians to 54% in the last available Australian Bureau of Statistics survey ([9](#_ENREF_9)), is another important current driver of the increasing numbers of patients entering cirrhosis and CLD.

**Problems with the current management models of CLD**

A number of effective, evidence-based treatments have been shown to improve survival in CLD patients. These include disease specific therapies such as; alcohol abstinence for alcoholic liver disease, antiviral therapy for viral hepatitis and immunosuppressive therapy for autoimmune hepatitis. In addition a number of evidence based therapies can improve survival for end stage manifestations of CLD including; antibiotic prophylaxis for patients with ascites, primary prophylaxis using beta blockers or endoscopic variceal ligation for high risk varices, surveillance for HCC in at risk patients and aggressive nutrition of severely malnourished cirrhotic patients. Large volume paracentesis, sodium restriction and diuretics for ascites can improve patient quality of life and reduce hospitalization ([10](#_ENREF_10)) as can available treatments (lactulose and rifaximin) for hepatic encephalopathy ([11](#_ENREF_11)). For patients who fail to improve despite medical therapies, liver transplantation can restore normal liver function and is associated with an overall 80% 5-year survival in Australia and New Zealand.

However, despite the availability of effective therapies, available data suggests the delivery of such care to CLD patients is suboptimal. In routine practice poor rates of variceal screening and prophylaxis of high risk varices ([12](#_ENREF_12)), poor adherence to guidelines for hepatocellular carcinoma surveillance ([13](#_ENREF_13), [14](#_ENREF_14)) and poor adherence to ascites management guidelines ([15](#_ENREF_15)) have been observed by multiple investigators. Suboptimal care is a likely contributor to frequent, prolonged, costly and often preventable readmissions that characterize this disorder ([16](#_ENREF_16)). The CLD-related readmission rates have been identified as 14 %, 37 % and 53 % at 1 week, 3 months and 6 months respectively by recent studies ([16](#_ENREF_16), [17](#_ENREF_17)), with an estimated 52 % of these readmissions judged as preventable by improved care. The reasons for inadequate care delivery in CLD patients appear complex and multi-factorial. The lack of hospital clinician focus on preventative care measures, absence of robust systems to improve patient follow up and monitoring following discharge, and poor integration of hospital and primary care team members are all likely contributing factors. Finally, poor engagement of patients in their own care and inadequate patient education is also likely to be of major importance.

**Chronic Disease Management Models**

Significant changes in the current model of care are required in order to make meaningful improvements in the management of this complex and challenging disease. A candidate model is the CDM model, first articulated by Wagner et al. in 1996 ([18](#_ENREF_18)) and subsequently developed by other groups. The key components of CDM are; self-management support, delivery system design, decision support and clinical information systems, community linkages and support from health care organizations. CDM approaches have produced positive outcomes in a number of non-liver chronic disease settings including; heart failure, ischemic heart disease, chronic obstructive lung disease and diabetes ([19](#_ENREF_19)). Three meta-analyses of over 30 randomized controlled trials (RCTs) using a variety of CDM approaches in heart failure have shown similar results. These studies demonstrated a 30% to 42% reduction of heart failure related admissions and a 12% to 27% reduction in all cause admissions, together with a mortality reduction of 18% to 25 % relative to usual care ([20-22](#_ENREF_20)). These clinical improvements were also associated with significant cost savings in 15 of the 18 trials where financial outcomes were evaluated ([20](#_ENREF_20)). As a result of these studies CDM style approaches are now the standard of care in heart failure and have been given a class 1 recommendation in practice guidelines ([23](#_ENREF_23)). Similar findings have been reported for chronic obstructive lung disease with a meta-analysis of RCTs of CDM programs demonstrating a 22% reduction in hospitalizations, 42% reduction in unscheduled emergency and outpatient visits, but no reductions in mortality ([24](#_ENREF_24)).

**Preliminary studies of CDM models in CLD**

CLD would appear to be a logical choice for CDM style interventions. It is characterized by frequent, lengthy and costly admissions and has effective but poorly implemented evidence-based treatment. However, it remains unclear if findings from other disease trials can be adapted to the unique challenges of CLD which include; high short term mortality, high rates of drug and alcohol addiction, frequently social disadvantage and marginalization from health care and multiple aetiologies and clinical presentations. The urgent need for RCTs has been highlighted by editorials in high impact gastroenterology journals ([25](#_ENREF_25), [26](#_ENREF_26)). However, the only RCT published to date has been a pilot trial performed by Wigg et al ([27](#_ENREF_27)) and subsequently editorialized by high impact gastroenterology journals ([28](#_ENREF_28), [29](#_ENREF_29)). In this single centre trial of 60 patients a multifaceted CDM model was used during a 12 month period. The main components of this intervention were delivery system re-design (co-ordinated case-management, home visits, weekly telephone reviews, rapid access to care pathways) and increase self-management support (patient action plans, increased education and self-monitoring, medication blister packs).

The major findings of this pilot trial were:

- No reduction in the primary endpoint of liver-related occupied bed day rates in the intervention versus usual care arms (17.8 vs 11.0 bed days/person/year, incidence rate ratio=1.6, 95% CI=0.5-4.8, P=0.39), however a non-significant 21 % reduction in unplanned/emergency admissions
- Non-significant reduction in mortality in intervention arm (Hazard ratio 0.6, 95% CI=0.3-1.5, P=0.32)
- Significantly improved quality of life for intervention patients relative to baseline but no significant improvement relative to usual care group
- Significantly improved quality of care in intervention group for outpatient attendance and adherence to protocols for HCC screening, vaccination and bone density surveillance.
- Cost effectiveness was demonstrated in a subsequent analysis which showed the intervention was less expensive per patient by a mean of $ 57,000 per participant with a 96 % probability of cost effectiveness at a willingness to pay of $ 40,000 per death avoided ([30](#_ENREF_30)).

A further published trial investigating the effects of CDM/care co-ordination models in cirrhosis was a non-randomized study of 100 patients followed for 12 months ([31](#_ENREF_31)). This model consisted of a “care management check-up” whereby all investigations were performed and preventative treatments commenced during a day admission to a day hospital.

The chief findings of this study were:

- Significantly reduced mortality in the intervention group (23% versus 45.7%, p<0.025)
- Significantly reduced 30-day readmission in intervention group (15.4% vs. 42.4%, p=<0.01)
- Significantly lower cost per patient month in the intervention group (1479€ vs. 2816€, p<0.05)

A final non-randomized study by Tapper et al ([32](#_ENREF_32)) investigated prospectively the effects of an electronic discharge checklist on subsequent readmission rates in patients with cirrhosis. The electronic decision support significantly reduced readmissions by 40%, mainly driven by improved compliance with rifaximin and reduced encephalopathy related readmissions.

**Indication for proposed new trial**

Providing a firm evidence base for CDM models in cirrhosis is one of the key research priorities currently in gastroenterology due to the rising prevalence and costs of CLD and was a key recommendation of the recent report commissioned by the Gastroenterology Society of Australia ([2](#_ENREF_2)). Unfortunately the current evidence base for CDM models in CLD, described above, is inadequate to support the use of these models in CLD.

While the pilot study of Wigg et al was an informative study, limitations of the study also limit its clinical usefulness. Firstly the study was designed to investigate feasibility of a future RCT and was not powered to investigate the most clinically relevant endpoints of patient mortality and cost effectiveness. Secondly the small sample size of the pilot trial left it susceptible to imbalance of disease aetiology between groups, with a higher proportion of alcoholic liver disease in the usual care group. A further concern was the trial setting in a single, tertiary care, transplant centre and high quality of care observed in the usual care group may have concealed benefits of the intervention that may have been revealed with a multiple site investigation. A requirement for hospital admission for trial entry may have also introduced a selection bias of terminal phase patients, too sick to benefit from the intervention The relatively short 12-month follow up of the trial may also not have captured the longer-term beneficial effects of the intervention. The unexpected trend towards increased hospitalization also suggests that CDM style interventions may have different effects in CLD compared with other chronic diseases with increased admissions of relatively sicker patients due to improved access to care. This phenomenon has previously been noted in primary health care settings treating patients with severe chronic diseases ([33](#_ENREF_33)).

Conclusions from the aforementioned “care management check-up” study ([31](#_ENREF_31)), while promising, are also limited by significant flaws in study design. Lack of randomization, lack of process measure measurement, unexpectedly high death rate in usual care group, use of adjusted rather than unadjusted analyses and failure to provide robust cost effectiveness data are all significant concerns that have been highlighted ([26](#_ENREF_26)). While the Tapper study ([32](#_ENREF_32)) also shows the potential of electronic decision support interventions conclusions and are also limited by its lack of randomization and generalizability is limited by the use of sophisticated electronic medical records and the study setting in an advanced quaternary care transplant unit.

In summary appropriately powered and designed trials of CDM in CLD with appropriate endpoints are an urgent and important research priority in gastroenterology to help provide definitive evidence concerning their effectiveness and cost effectiveness to both clinicians and funders of healthcare. The proposed larger trial will address weaknesses of the pilot trial via; modification of the intervention, increased power and follow up time, reduced severity of liver failure, increased generalizability of results due to multicentre setting and multiple CLD nurses.

# Study Objectives

## Primary Objective

To investigate changes in hospitalization rates associated with the intervention. Specific measures of hospital usage will include liver-related (unplanned and planned) admission and occupied bed day rates, all cause admission and occupied bed day rates, intensive care admission and occupied bed day rates and median length of stay.

## Secondary Objectives

- To evaluate incremental cost effectiveness for this intervention, relative to usual care. Assessment of quality of life will be a necessary component of the economic analysis. The QALY will be used to assess this outcome.
- To evaluate the effect of CDM on mortality
- To evaluate the effect of CDM on the quality of care provided to patients in the intervention arm. This will be assessed using the following measures:
  - attendance at planned outpatient care;
  - medication adherence;
  - adherence to antibiotic prophylaxis protocols for patients with ascites;
  - adherence to variceal bleeding prevention protocols;
  - adherence and response to alcohol abuse treatment protocols in alcoholic liver disease patients; and
  - referral rates to liver transplantation assessment teams.
- To compare self-management ability and knowledge of patients in the CDM arm with usual care models.

# Research Plan and Methods

## Trial design

The planned study will be a multi-centre, randomized controlled, parallel-group trial with 1:1 allocation ratio of a CDM model intervention versus usual care in CLD patients. The study will follow CONSORT guidelines, where relevant, for the design, conduct, and analysis of RCTs.

## Study duration

At each site, recruitment will occur for 12-18 months, and then the trial will continue until the last participant recruited has had 24 months of CDM (total 3-year study duration).

## Number of Participants

The aim is to enrol 150 patients into the study but sites will be allowed to recruit until a maximum of 200 patients are enrolled.

## Treatment Assignment

Participants will be randomized in a 1:1 ratio to receive usual care or the intervention from Day 1 through to study end.

## Participant Population

This study will be conducted at 4 sites in Australia at which the CDM model of caring for patients with cirrhosis has not been instigated. Participants will be from inpatient admissions where patients have come into hospital with a symptom of decompensation.

## Participant Inclusion Criteria

1. Cirrhosis of the liver
2. Admitted to hospital with an episode of decompensation including one of:
   - Ascites
   - Encephalopathy
   - Variceal bleed
   - Spontaneous bacterial peritonitis
   - Hepatorenal syndrome
3. Patients who have attended an outpatient liver clinic with a hospital admission in the last 6 months and a Child Pugh B or C score
4. Able to comprehend and willing to sign an Informed Consent Form (ICF) (West Haven Criteria = 0 or 1)
5. Patients with mild cognitive impairment, at PI’s discretion
6. Age ≥18 years

## Participant Exclusion Criteria

1. Currently involved in a multidisciplinary heart failure program
2. Diagnosis of active hepatocellular carcinoma (HCC)
3. Active management by Palliative Care Services or expected survival of < 3 months
4. Currently on the liver transplant waiting list

## Consent of potential participants

Consulting clinicians, or the study PI, will verbally consent patients to allow the study team to access their medical records to determine eligibility into the study. Consent will be recorded either electronically or in hardcopy patient notes. Upon consent, study nurses will review case notes to determine potential inclusion. The treating team can also let the trial nurse know if there is a potential participant under their care. If Hepatic Encephalopathy is the admission criteria or a present as a concurrent condition on admission the investigator needs to provide an assessment for Encephalopathy using a standard assessment – the West Haven Criteria. A score of 0 or 1 documented in the patient notes by the treating team or trial investigator means that the patient can be approached for consent. Participants who meet inclusion criteria and do not have exclusion criteria will be approached by the site investigator or site nurse. Investigators or nurses can obtain consent for the trial. Potential participants will be inpatients in hospital, or approached after an outpatient appointment, and will receive the information sheet with an explanation about the trial by investigators or nurses and be given opportunity to discuss the trial with family members or to consider the trial. Consent will occur before the patient is discharged from the hospital and can be obtained by investigators or trial nurses. Both consent for the ALFIE trial as well as specific consent for access to MBS and PBS data will be sought. The intervention will commence prior to discharge from hospital.

Patients with mild cognitive impairment may be approached and consented for the study at the site PI’s discretion. If required, a third-party person responsible PISCF can be provided by the PI or study coordinator to the person responsible for the potential participant to obtain informed consent.

The patient will be reassured verbally that their medical care in hospital will not be affected by their participation or non-participation in the trial.

*Recruitment during the COVID-19 Pandemic*

Due to the COVID-19 pandemic and the need to maintain social distance from all potential participants, recruitment methods will be altered to ensure patient and staff safety.

Potential participants admitted to hospital may be approached by the site CLD nurse, or if restrictions are in place, the treating doctor or site PI can approach the patient about the study and undertake consenting as per current protocol.

Participants that would usually be approached during an outpatient appointment may now be contacted about the study via a phone call from the CLD nurse. The nurse will explain the study to the patient as per usual process. If the patient is agreeable, then the CLD nurse will email the patient using the ALFIE Email Consent Template. The email, with site PI cc’d, will contain details about the trial, and will have the ALFIE PISCF, the ALFIE MBS/PBS Consent Form and baseline electronic surveys attached. The patient will be asked to reply to both CLD nurse and site PI confirming that they have read and understood the PISCF and convey that they consent to taking part in the trial. Consenting participants will need to also convey consent to MBS and PBS data access, as well as send back the completed electronic surveys. The CLD nurse will then print the email consent and file into site folders and continue to randomisation. Hardcopies of the consent forms will be sent to each consenting patient with a reply paid envelope as Services Australia requires the hardcopy consent to release MBS and PBS data.

# Intervention and Implementation

The term ‘intervention’ refers to the Chronic Disease Management model applied to the care of the patient with cirrhosis of the liver. The intervention starts after randomisation and will continue until the end of the study which is 24 months after the first randomised patient.

Randomisation signals the start of the treatment period. Every Participant will have at 24 months of intervention.

## The Chronic Disease Management Model:

- A ‘patient identification card’ (Appendix B) for patients to use to indicate their involvement in liver disease case management to use in emergency departments, GPs, or other health services.
- An initial home visit(s) for assessment and education within 7 days of discharge from hospital or consent and includes education for:
  - Medication adherence
  - Cirrhosis education
  - Assessment for complications of liver disease not previously diagnosed
  - Assessment of any barriers to self-management
- Development of a Care Plan with the Hepatologist and the patient
- Weekly phone contact for the first 3 months of intervention for ongoing assessment and education, then 3 monthly to 12 months, then 6 monthly if appropriate.
- Early Hepatologist review post hospital discharge (within 4 weeks)
- Patient reminders prior to appointments
- Case management of symptoms of decompensation such as ascites, hepatic encephalopathy and SBP which are protocol led
- Referral pathways for alcoholic liver disease
- General Practitioner communication including entry into the study, discharge summaries, care plans, changes to medications, and discharge from the service.
- A direct telephone number provides rapid access to care for patients requiring symptom assessment and management
- Case conference reviews during hepatology outpatient appointments attended by the patient and the CLD nurse

**Decision making support is available to the CLD nurse via:**

- Evidence based protocols for all major complications of cirrhosis provided to all CLD nurses
- Protocol driven CLD and disease specific checklist

**Self-management support will be given to the patient via:**

- - Patient information booklet on cirrhosis containing medication list, weights, action plans, appointment details, contact numbers
  - Enhanced patient and carer education during contacts with the CLD nurses concerning diet, medications, need for investigations
  - Self-monitoring of weight for patients with ascites using digital scales
  - Medication blister packs where applicable to improve adherence

**Clinical information systems:**

- Individual patient data sheets with problem lists, medication lists and weight charts provided by CLD nurses to hepatologists during outpatient reviews.
- Recall and reminder system for CLD nurses using Microsoft office.

## Role of CLD nurse

Patients in the Intervention Group (Case Management (CM) group) will have a CLD nurse home assessment within a week of randomization. This assessment, together with input from the specialist will produce a protocol driven, but individualized care plan which will be distributed by the CLD nurse to the patient, gastroenterologist and GP and be placed in the patient’s hospital record. The CLD nurse will then facilitate implementation of the plan by promptly organizing required investigations and assisting the patient to attend and coordinate their own medical and allied health visits, and provide written ± telephone reminders prior to each liver-related appointment. Patients in the CM group will be reviewed by CLD nurses through weekly telephone calls. Patient deterioration will be promptly reported to the patient’s specialist, to enable early intervention and help avoid emergency admissions. Patients who achieve stable clinical states by three months may subsequently receive less intense monitoring with telephone contact as designated by the treating team, which can be escalated as required. CLD nurses will support the patient and carer in their communication with the health care team and will educate all those involved in the care plan about the model of care and the central role of the patient. CLD nurses will be accessible to patients, carers and team members via mobile phone during business hours. For after-hours medical advice the CLD patients will access on call teams for each site via usual care pathways.

## Role of Gastroenterologist/Hepatologist

Gastroenterologists usually involved in the inpatient and outpatient care of CLD patients at each site will continue to be involved with both intervention and usual care patients. However, the CLD nurse will improve the continuity of care with gastroenterologists for intervention patients by facilitating optimal patient contact with their usual gastroenterologist during the trial. The gastroenterologists involved with intervention patients at each site will be supported by the dedicated Hepatology chief investigator at each site. The gastroenterologist will be involved in formulation of the initial care plan and subsequent modification when appropriate. The gastroenterologist will be accessible to the CLD nurse for non-contact patient reviews when concerns arise about patient deterioration. The gastroenterologist will receive enhanced support during non-contact and direct contact patient reviews from the CLD nurse who will provide care plan updates which include: medically defined problem lists, medication lists, relevant clinical data (i.e. MELD scores and weight charts), key investigation results and patient defined problem lists including personal and lifestyle goals.

## Role of General Practitioner

Following recruitment, the CLD nurse will involve the intervention patient’s GP at an early stage and will communicate hospital information as well as care plans from the intervention. GP communication will occur (using a method chosen by the patient’s GP) at hospital admission or discharge, or any major change to the patient’s care plan.

## Control/usual care group

Control/usual care patients will continue hospital and out of hospital management via usual care processes. To prevent contamination of usual care patients by the intervention, usual care patients will have no contact with CLD nurses until a final end of study interview. Usual care patients will be mailed surveys at baseline, 3 months, 6 months, and then 6-monthly until the end of the trial (24 months after the first patient randomised). At the end of the trial, control group participants are invited for a face-to-face review to complete the surveys, are given the Cirrhosis Booklet and offered an education session.

## Adverse Events

Any untoward medical occurrence in a patient, which does have a causal relationship with the treatment intervention, is considered an adverse event (AE).

Examples of AEs possible in this study include (but are not limited to): stress and/or anxiety induced by the study surveys; anxiety caused by contacting a deceased patient’s family.

# Treatment of Participants

## Concomitant medications

Relevant information about all concomitant medications (Including prescribed, over-the-counter, or herbal preparations) taken from the start of the study to the end of the study must be recorded on the source document ‘concomitant medications’ (and submitted to for data entry at baseline and at the end of the trial.

## Randomisation

The study will be conducted in a randomised controlled manner. Allocation to one of two treatment groups (intervention or control) will occur on a 1:1 ratio across sites and will be stratified by aetiology. A randomisation list will be generated by a statistician and allocations, stratified by aetiology, will be placed into sealed opaques envelopes until randomisation. Upon consent, the CLD nurse will contact an independent researcher at FMC for telephone randomisation. Participant allocation, using unique study ID, will be recorded by the independent researcher and stored in a secure cabinet.

## Assignment of Site and Participant Numbers

Each study site will be assigned a site number by the coordinating site. The site number will be used to categorize Participant data and to identify the site and/or investigator within study documents. Participants will be identified using a unique 4 digit number. The first digit will represent the site number and the last three digit will represent the patient number. The patients are given sequential numbers (ie at each site the first patient will have number 001, then the second 002 etc).

# Visit Procedures

## Pre-Screening

Patients who are admitted with a liver-related issue will be assessed for eligibility for the study. Inclusion and Exclusion criteria are applied and patients who meet all criteria call be approached for consent. Patients with any diagnosis of Hepatic Encephalopathy need to have a documented West Haven Criteria Score of “0” before they are approached.

## Baseline

Baseline visits will begin after

- - The signature of the informed consent form (ICF)
  - The study number is allocated (Site Number-XXX)

### Baseline – all patients

Patients in the intervention and control group will have the following collected using the Baseline collection tool (Appendix A):

- - Demographics
  - Aetiology of cirrhosis
  - Child Pugh/MELD Score
  - All surveys (PIH, CLDQ, Cirrhosis Knowledge Assessment Questionnaire, EQ-5D-5L, ASK-12, and resource use survey)
  - Concomitant medication list (as per current hospital medication chart)
  - Comorbidities list and the Charlson Comorbidity index

Once all of the baseline information is collected Randomisation occurs via the telephone contact.

If a patient has been randomised to receive usual care, please advise them that they will be receiving future surveys by mail and that at the end of the trial they will be invited to come in for a face to face meeting and education. Ensure their address and contact details are correct.

### Baseline – intervention only

After randomisation, patients in the intervention group continue to have information collected which helps with the development of their care plan. It is best to collect this information as soon as possible after randomisation – but within 7 days of randomisation.

- GP communication informing of the patient’s involvement (see fax templates)
- Make appointment for home visit (if discharge date known) – complete safety check for home visit
- Ascites and HE assessment
- HCC assessment
- Osteoporosis assessment
- Alcohol assessment
- Variceal screening assessment
- Nutrition assessment
- Carer and GP assessment

The care plan can start to be developed at this point. Ensure that once the patient has discharged from hospital, they have an outpatient review by a gastroenterologist/Hepatologist scheduled within a month (4 weeks) of discharge. Please provide the patient with a participant identification card. Contact the GP by phone to establish accepted communication methods and communicate hospital discharge information.

## Home Visit

Intervention participants will have a home visit from the trial nurse booked within 7 days of hospital discharge. At this point the care plan is further discussed with the patient and interventions agreed upon with the patient. The home visit is an opportunity to increase rapport and develop your clinical relationship and trust, to assess patient risk, and assess their physical state and symptoms after discharge from hospital. Although some issues such as medication management can start to be addressed in hospital, it is only at the home visit that the nurse can really assess how the participant manages their medications. The home visit will only be provided to patients in the Metropolitan area.

Prior to the home visit, ensure you complete your site’s home visit risk assessment form as local protocol requires. If the risk assessment shows unacceptable risk the visit is not to take place and the home visit procedures conducted over the phone.

If risk is acceptable, at the home visit, complete the ALFIE Study home visit form. It is envisaged that the home visit will take 30-60 minutes. Time the visit and enter the duration of the visit on the Home Visit form (Appendix I). Complete a full report of the visit in the participant’s case note as a record of your findings and to record the plan.

## Telephone Contact

Patients in the intervention group receive (at least) weekly telephone calls for the first 3 months. Ensure all patient contacts are recorded in the patient contact log (Appendix M). There is a window of +/- 2 days for all patient contacts.

## Outpatient review by Specialist Gastroenterologist/Hepatologist

Review by a specialist must be scheduled within 1 month (4 weeks) of discharge from hospital for all patients in the intervention group. The nurse must attend this appointment with the patient. In preparation for the outpatient appointment the following should be updated:

- - Medication list
  - Care Plan
  - Ensure screening and surveillance requirements are up to date (or booked)

At this appointment the specialist will discuss the care plan with the nurse and the patient and sign off on the care plan.

## Month 3, Month 6, Month 12, Month 18

Patients in the intervention group have their Child Pugh and Meld scores completed at 3, 6, 12, 18 months and at end of trial (Appendix N). Collect the information from the most recent bloods taken as part of usual care, the patient will not be required to have a blood test taken just for this score to be done.

Surveys are due for both groups at this time. For intervention patients this should ideally be scheduled at the same time the specialist outpatient appointment is scheduled to enable accurate determination of the Child Pugh and MELD score. Ensure the contact log is up to date. Patients will be provided the option of receiving the surveys via standard post or electronically, via email, at consent.

Record hospital and liver-related outpatient admissions/attendances/non attendances at the month 12 visit.

## End of Study

Patients will have an end of study visit at withdrawal, when listed on the transplant waiting list, or at the end of the study (24 months after the randomisation of the first patient).

Both groups will have the following collected:

- Cirrhosis Knowledge Assessment Questionnaire
- Ask 12
- CLDQ
- EQ-5L-5D
- PIH Scale
- Patient Satisfaction Questionnaire
- Resource use questionnaire
- MBS and PBS information (to be collected centrally by the coordinating site) if the patient signed the consent form at the beginning of the trial.

Please remind the participant that a member of the study coordinator team may be in contact via telephone to ask a few questions about their experiences but they can refuse to participate if they wish. Thirty two patients will be consecutively recruited (8 patients per site, 4 patients from each study arm) for the telephone interviews.

The following will also be completed/updated at end of study in both groups:

- Hospital admissions log
- Outpatient attendance log
- Concomitant medications
- Comorbidities list
- Child Pugh & MELD score
- HCC assessment
- Ascites and HE assessment
- Osteoporosis assessment
- Alcohol assessment
- Variceal assessment
- Nutrition assessment

It is preferable that the information is collected face-to-face as the control group patients can have some education about their liver disease if they wish. Ensure they receive the Cirrhosis Booklet.

# Qualitative Analysis : Patients and Staff

A qualitative analysis of the patients’, nurses’ and doctors’ perceptions of the ALFIE trial is essential to assess the efficacy of the trial and to improve the delivery of the intervention according to their needs.

The primary aim of this qualitative review is to understand more about the actual conduct of the trial, the issues, challenges and learnings; to understand how and why the implementation achieved what it did. Secondary aims are to:

1. Analyse the patients’ experience in the ALFIE trial from the perspective of patient satisfaction, education and facilitation of self-management.
2. To determine the satisfaction of the CLD nurses and hepatologists involved in implementing the intervention.

An inductive, descriptive qualitative study design with qualitative interviews has been chosen for this qualitative review.

## Number of participants

Thirty two patients will be consecutively recruited over two five month periods — October 2020 to February 2021 (block 1) and October 2021 to February 2022 (block 2). Each site will aim to recruit 8 patients in total, with 4 patients from each study arm.

Seven nurses and five hepatologists will be approached for participation at the end of the study.

## Recruitment and consent process

Patients: Informed consent for the interviews was obtained during consent into the study, but patients will be provided the option of being contacted or not. Potential participants will be invited to take part in the telephone interview by their CLD nurse during their end of study visit and will obtain consent for the designated interviewer from FMC to contact them. The CLD nurse will inform the patient that the interview will take approximately 20 minutes and will involve briefly talking about their experience being involved in the ALFIE trial. If the patient consents, the CLD nurse will pass on their name and contact details to the designated interviewer. The designated interviewer from FMC will then contact the patient to conduct the interview. The participant will be advised that they can change their mind and cancel the interview if they wish.

Staff: The designated interviewer from FMC will email staff participants with the information sheet and request a convenient time to conduct the interview. As all interviews will be conducted over the phone, the staff participant will respond with email consent if they are interested in participating and will have seven days to consider participation. Upon consent, staff participants will be provided the interview questions 1 day prior to the interview, to ensure that they have sufficient time to consider their answers. The participant will be advised that they can change their mind and cancel the interview if they wish.

## Data collection, storage and analysis

All interviews will be conducted over the phone. The interview will be audio-recorded and transcribed verbatim, and field notes will be taken during and after each interview. One researcher, a registered nurse and not part of the core research team, will conduct all the interviews. The designated interviewer will be provided a set of standardised questions to ask the patients (Appendix V) and staff (Appendix W) during the interview. The interviews will be open ended and carried out as a conversation to understand the participant's experience with the ALFIE trial. For the patient interviews, the designated interviewer will be blinded to the patient’s randomisation group and treating hospital.

Recordings of interviews will be allocated a unique study ID prior to being sent for transcription. All data will be assigned a unique study ID and will be stored securely, in a password protected computer and only FMC study investigators will have access to the data. Hardcopies of the transcriptions will be kept in a secure locked filing cabinet in the Hepatology Department research office at FMC. Participants will be given the option of requesting the transcript to check their answers.

A qualitative approach will be used to analyse the collected data as described in grounded theory. This approach for content analysis is interpretive in nature and used to describe or illuminate a phenomenon through identification of manifest (the obvious) and latent (underlying meaning) content in a text. This will involve: (1) reading of the text several times to become familiar with it and reflect upon the content, (2) identification in the text of meaning units that describe the phenomenon, (3) meaning units condensed and essential content is abstracted and labelled with a code, and (4) codes compared based on similarities and differences sorted into themes. Coding and sorting of themes will be assisted using the analysis took NVivo.

# Outcomes

## Unplanned liver-related hospital admission rate

Unplanned liver-related hospital admission rate will be the primary outcome measure of the trial. Other hospitalization measures, as outlined in the Aims section, will be measured as secondary endpoints.

## Incremental cost effectiveness relative to usual care and quality of life

The economic evaluation will be carried out to assess the incremental cost effectiveness of the CDM model, relative to usual care, for patients with decompensated cirrhosis over the study duration. The primary economic outcome will be the incremental cost per unplanned admission avoided in the intervention arm relative to the usual care arm. Secondary economic outcomes will include the incremental cost per death prevented and incremental costs per quality adjusted life year (QALY) gained. QALYs will be based on responses to the EuroQoL 5 dimensions 5 level (EQ5D 5L) and a validated disease specific Chronic Liver Disease Questionnaire (CLDQ)([43](#_ENREF_43)).

Incremental costs associated with the intervention compared to usual care over the study period will be estimated using Medical Benefits Schedule (MBS) and Pharmaceutical Benefits Schedule (PBS) data to estimate primary care, pharmaceutical, and private hospital costs, and centralised costing data for inpatient episodes at public hospitals (Australian Refined Diagnosis Related Groups (AR-DRGs) cost weights). Consent to access PBS/MBS/AR-DRG data will be sought from recruited patients. Intervention specific costs (e.g. labour costs, travel and telephone expenditure etc) will be collected administratively within the trial. Patient level measures of utility derived from the EQ-5D 5L and CLDQ instruments (measured at baseline 3 and 6 months then 6-monthly in the intervention group and at the end of trial in both groups) will be integrated with survival curves in order to calculate QALYs over the study period. This economic evaluation will include cost-effectiveness, acceptability, net benefit and expected net loss curves to inform decision makers of the optimal strategy at any given threshold, and uncertainty around this decision.

At the end of the study, both groups will be asked to complete a short resource use questionnaire asking patients to share how much time and money have been spent on health care over the past 2 weeks.

## Mortality and disease severity

Mortality at end of study follow up will be a further secondary endpoint of the study. To ensure patients lost to follow up have not died, cross checking with Australian death registries will occur. Other clinically relevant indicators of liver disease severity MELD and Child Pugh score, will also be monitored at baseline, study end and 3-monthly during the trial period.

## Quality of care indicators

Quality of care indicators, as outlined in the Aims section, will be measured. These indicators are recognized quality indicators ([44](#_ENREF_44)) and highly relevant process measures of successful implementation and effectiveness of the intervention. Attendances will include all planned outpatient medical, allied health, radiology and endoscopy appointments.

## Patient Satisfaction and Self-Management Ability

The following surveys will be administered at baseline, 3 months, 6 months and then 6-monthly and at the end of the trial:

- PIH - The Partners in Health (PIH) scale to assess changes in patient self-management ability ([45](#_ENREF_45)).
- A local disease specific knowledge assessment questionnaire, the “Cirrhosis Knowledge Assessment Questionnaire”.
- The ASK-12, a medication adherence tool will be used to determine medication adherence
- A Satisfaction Survey will be administered at the end of the study in both groups.

Qualitative feedback from patients will also be sought at the end of trial via a telephone interview with a researcher not directly involved in the patient’s care. Although consent for inclusion in the interview will be sought at the start of the trial, patients will be given the chance to ‘opt out’ if they are approached to participate and decide that they do not wish to. After the interview if patients feel they wish to change their answer or review their answer, a number will be provided so they can do so within a certain time.

## Intervention period

Patient recruitment will occur during a 12-18 month window and the intervention will occur for 24 months for each patient. In the case of patient death, liver transplantation and loss to follow up, the study will end.

# Statistical Methods

## Sample size and power analysis

The trial will be powered for the primary endpoint of unplanned liver-related admissions. Assuming an admission rate of three admissions per person per year in the usual care group, and a reduction of 20 % in the treatment group, a sample size of 146 (50 per site) would provide 80% power to detect a significant difference in event rates between groups at a type 1 error rate of α=0.05. This assumes a median follow-up time of 18 months, as well as allowing for a 20% attrition rate throughout the trial. Recruitment of a sample size of 147 patients within 12 months is feasible and represents approximately 38 patients per site. The anticipated caseload of intervention patients per site is 19, which was less than the caseload during the pilot trial (40) and will be feasible with a 0.6 fte RN level 3 nurse at each site.

## Randomization, stratification, blinding

Following informed consent, randomization (75 patients in each arm) will occur via telephone call to a third party (Flinders Medical Centre Research Department, Adelaide), with subsequent selection of opaque, sealed envelopes containing the treatment arm allocation number. To prevent imbalance in disease aetiology, stratified block randomization will be performed for disease aetiology. Strata for alcohol, hepatitis C and “other causes” will occur. With three strata and block sizes of four, the maximum imbalance between treatment groups at the end of randomization will be six, which will not significantly affect statistical power. Blinding of care providers (trial nurses and gastroenterologists) will not be possible with this trial design but outcome assessors will be blinded.

## Statistical methods

All analyses will be performed according to an intention-to-treat protocol. Differences between groups for hospitalization rates, quality indicators, satisfaction rates and self-management scores will be assessed using negative binomial regression, which is appropriate for count data with evidence of over-dispersion. All models will allow adjustment for baseline values and other co-variates. Suitable transformations will be applied in the case of non-normally distributed data.

For the economic analysis mean costs between the intervention and control groups will be compared and incremental cost effectiveness ratios presented with confidence intervals. Cost effectiveness acceptability curves for varying threshold values of cost effectiveness will also be presented ([46](#_ENREF_46)). An assessment of the sensitivity of the results obtained to variation in measured resource use, effectiveness and/or unit costs will be undertaken using appropriate one-way and multi-way sensitivity analysis ([47](#_ENREF_47)). Differences between groups in quality of life scores will be assessed using mixed effects regression to account for the repeated measures (every 3 months) and subsequent within-Participant correlation. Survival analysis will be performed using Cox regression with results presented as Kaplan-Meir survival curves.

# Trial Management

## Training

To optimize implementation, a two-day training workshop will be held for the four CLD nurses employed by the trial. The workshop will be run by Alan Wigg, Rosemary McCormick and Rachel Wundke, who developed extensive experience in CLD CDM practice during the pilot. The aims of the workshop will be to familiarize nurses with CLD medical management protocols, standardize nursing approaches to CDM, and research processes and data collection requirements. Data entry will be centralised and double key verification will be included. Consistency checks will be incorporated into the data entry procedure. Principal Investigators from each site will also join for a morning of training about the study requirements, medical protocols and data collection requirements.

All investigators will teleconference initially monthly to oversee quality management and timeline issues related to the project. In addition, a Project Management Executive involved in the day-to-day management (Alan Wigg, Jeyamani Ramachandran, Rachel Wundke and CLD nurses at SCGH, QEH, LMH, and RAH) will initially teleconference weekly for the first two months, then fortnightly-monthly throughout the duration of the project. The trial managers (Rachel Wundke) will be responsible for operational management of the trial and Libby Bambacas will be responsible for data entry and randomisation and will remain separate from day-to-day management.

## Data Monitoring

Outcome data for all endpoints will be monitored at 3-monthly intervals during the trial by the project team and at the trial midpoint to review adverse events, progress and preliminary outcome data.

Data will be submitted by scanning data collection records to the trial data manager. If a patient consents to receiving study surveys electronically, the CLD nurse involved in their care will send fillable surveys at each time point to the provided email address. The surveys will have the patient’s unique study ID allocated. Completed surveys will be emailed back to the CLD nurse, who will then check to ensure there is no identifying information recorded, before forwarding data on to the trial data manager as per standard process. All data will be saved in a secure password-protected computer for the duration of the study, which can only be accessed by the trial data manager.

| Activity Schedule for CONTROL patients | Baseline | Treatment Period  Day 7 to Week 11 are scheduled from the discharge date from hospital.  From Month 3 visits are scheduled from randomisation.  All visits/contacts have a window of +/- 2 days | | | | | | | | | | | | | | | | |
| --- | --- | --- | --- | --- | --- | --- | --- | --- | --- | --- | --- | --- | --- | --- | --- | --- | --- | --- |
|  | Baseline | Day 7 | Day 14 | Week  3 | Week  4 | Week  5 | Week  6 | Week  7 | Week  8 | Week  9 | Week  10 | Week  11 | Month  3 | Month  6 | Month  9 | Month  **12** | Month  18 | Month 24/EOS |
| Informed Consent | X |  |  |  |  |  |  |  |  |  |  |  |  |  |  |  |  |  |
| Child Pugh / MELD | X |  |  |  |  |  |  |  |  |  |  |  |  |  |  |  |  | X |
| SURVEYS (5) | X |  |  |  |  |  |  |  |  |  |  |  | X | X |  | X | X | X |
| Concomitant Medications | X |  |  |  |  |  |  |  |  |  |  |  |  |  |  |  |  | X |
| Comorbidities | X |  |  |  |  |  |  |  |  |  |  |  |  |  |  |  |  | X |
| Charlson Comorbidity Index | X |  |  |  |  |  |  |  |  |  |  |  |  |  |  |  |  |  |
| Randomisation | X |  |  |  |  |  |  |  |  |  |  |  |  |  |  |  |  |  |
| Cost/Resource use survey | X |  |  |  |  |  |  |  |  |  |  |  |  |  |  |  |  | X |
| Admissions to hospital |  |  |  |  |  |  |  |  |  |  |  |  |  |  |  | X |  | X |
| Liver-related appointments |  |  |  |  |  |  |  |  |  |  |  |  |  |  |  | X |  | X |
| Patient Satisfaction |  |  |  |  |  |  |  |  |  |  |  |  |  |  |  |  |  | X |
| Alcohol Use assessment |  |  |  |  |  |  |  |  |  |  |  |  |  |  |  |  |  | X |
| Ascites & HE assessment |  |  |  |  |  |  |  |  |  |  |  |  |  |  |  |  |  | X |
| HCC assessment |  |  |  |  |  |  |  |  |  |  |  |  |  |  |  |  |  | X |
| Osteoporosis assessment |  |  |  |  |  |  |  |  |  |  |  |  |  |  |  |  |  | X |
| Variceal assessment |  |  |  |  |  |  |  |  |  |  |  |  |  |  |  |  |  | X |
| Nutrition assessment |  |  |  |  |  |  |  |  |  |  |  |  |  |  |  |  |  | X |
| End of study outcome |  |  |  |  |  |  |  |  |  |  |  |  |  |  |  |  |  | X |
| Interview for selected pts |  |  |  |  |  |  |  |  |  |  |  |  |  |  |  |  |  | X |

| Activity Schedule for INTERVENTION patients |  | Treatment Period  Day 7 to Week 11 are scheduled from the discharge date from hospital.  From Month 3 visits are scheduled from randomisation. | | | | | | | | | | | | | | | |
| --- | --- | --- | --- | --- | --- | --- | --- | --- | --- | --- | --- | --- | --- | --- | --- | --- | --- |
|  | Baseline | Day 7 | Day 14 | Week  3 | Week  4 | Week  5 | Week  6 | Week  7 | Week  8 | Week  9 | Week  10 | Week  11 | Month  3 | Month  6 | Month  **12** | Month  18 | Month 24/EOS |
| Informed Consent | X |  |  |  |  |  |  |  |  |  |  |  |  |  |  |  |  |
| Child Pugh / MELD | X |  |  |  |  |  |  |  |  |  |  |  | X | X | X | X | X |
| SURVEYS (5) | X |  |  |  |  |  |  |  |  |  |  |  | X | X | X | X | X |
| Concomitant Medications | X |  |  |  |  |  |  |  |  |  |  |  |  |  |  |  | X |
| Comorbidities list | X |  |  |  |  |  |  |  |  |  |  |  |  |  |  |  | X |
| Charlson Comorbidity Index | X |  |  |  |  |  |  |  |  |  |  |  |  |  |  |  |  |
| Randomisation | X |  |  |  |  |  |  |  |  |  |  |  |  |  |  |  |  |
| Carer and GP assessment | X |  |  |  |  |  |  |  |  |  |  |  |  |  |  |  |  |
| GP communication^a^ | X | X |  |  | X |  |  |  |  |  |  |  |  |  |  |  |  |
| Face to Face Contact | X | X |  |  | X |  |  |  |  |  |  |  | X | X | X | X | X |
| Telephone Contact^b^ |  |  | X | X | X | X | X | X | X | X | X | X |  |  |  |  |  |
| Hepatologist OPD |  |  |  |  |  |  |  |  |  |  |  |  | X | X | X | X | X |
| Care Plan review^c^ | X | X |  |  | X |  |  |  |  |  |  |  | X | X | X | X | X |
| Alcohol Use assessment | X |  |  |  |  |  |  |  |  |  |  |  |  |  |  |  | X |
| Ascites & HE assessment | X |  |  |  |  |  |  |  |  |  |  |  |  |  |  |  | X |
| HCC assessment | X |  |  |  |  |  |  |  |  |  |  |  |  |  |  |  | X |
| Osteoporosis assessment | X |  |  |  |  |  |  |  |  |  |  |  |  |  |  |  | X |
|  |  |  |  |  |  |  |  |  |  |  |  |  |  |  |  |  |  |
| Activity |  | Treatment Period  Day 7 to Week 11 are scheduled from the discharge date from hospital. From Month 3 visits are scheduled from randomisation. | | | | | | | | | | | | | | | |
|  | Baseline | Day 7 | Day 14 | Week  3 | Week  4 | Week  5 | Week  6 | Week  7 | Week  8 | Week  9 | Week  10 | Week  11 | Month  3 | Month  6 | Month  **12** | Month  18 | Month 24/EOS |
| Variceal assessment | X |  |  |  |  |  |  |  |  |  |  |  |  |  |  |  | X |
| Nutrition assessment | X |  |  |  |  |  |  |  |  |  |  |  |  |  |  |  | X |
| Admissions to hospital |  |  |  |  |  |  |  |  |  |  |  |  |  |  | X |  | X |
| Liver-related appointments |  |  |  |  |  |  |  |  |  |  |  |  |  |  | X |  | X |
| Cost/Resource use survey | X |  |  |  |  |  |  |  |  |  |  |  |  |  |  |  | X |
| Patient Satisfaction |  |  |  |  |  |  |  |  |  |  |  |  |  |  |  |  | X |
| End of study outcome |  |  |  |  |  |  |  |  |  |  |  |  |  |  |  |  | X |
| Interview for selected pts |  |  |  |  |  |  |  |  |  |  |  |  |  |  |  |  | X |

a. GP Contact is to occur post discharge from any hospitalisation, post home visit, or any major change in the care plan. Please record all contact on the contact log.

b. If the nurse determines that a home assessment is required instead of a phonecall, this is allowed. Please document on the contact log. The contact specified is a minimum requirement. The nurse/Hepatologist can decide for more contacts than designated – please record on the contact log all patient contacts.

c. Care plan reviews include revisiting all care plan actions determined at baseline and ensuring all assessments, screening and surveillance and alcohol counselling are being actioned.

# References

1. Infante-Rivard C, Esnaola S, Villeneuve JP. Clinical and statistical validity of conventional prognostic factors in predicting short-term survival among cirrhotics. Hepatology. 1987;7(4):660-4.

2. Deloitte Access Economics; The economic cost and health burden of liver diseases in Australia. 2013.

3. 3303.0-Causes of Death in Australia. Australian Bureau of Statistics. In: Statistics ABo, editor. 2008.

4. AIHW National Hospital Morbidity Data [Internet]. 2010. Available from: [www.aihw.gov.au/hospitals/datacubes/index.cfm](http://www.aihw.gov.au/hospitals/datacubes/index.cfm).

5. 4832.0.55.001. Alcohol consumption in Australia: A Snapshot, 2004-05. Australian Bureau of Statistics. 2006.

6. Chikritzhs TN, Allsop SJ, Moodie AR, Hall WD. Per capita alcohol consumption in Australia: will the real trend please step forward? Medical Journal of Australia. 2010;193(10):594-7.

7. Liang L, Chikritzhs T, Pascal R, Binns CW. Mortality rate of alcoholic liver disease and risk of hospitalization for alcoholic cirrhosis, alcoholic hepatitis and alcoholic liver failure in Australia between 1993 and 2005. Internal Medicine Journal. 2011;41(1a):34-41.

8. Sievert W, Razavi H, Estes C, Thompson AJ, Zekry A, Roberts SK, et al. Enhanced antiviral treatment efficacy and uptake in preventing the rising burden of hepatitis C-related liver disease and costs in Australia. J Gastroenterol Hepatol. 2014;29 Suppl 1:1-9.

9. 47190.0 Overweight and obesity in Adults, Australia, 2004-05. Australian Bureau of Statistics. 2008.

10. Le S, Spelman T, Chong CP, Ha P, Sahhar L, Lim J, et al. Could Adherence to Quality of Care Indicators for Hospitalized Patients With Cirrhosis-Related Ascites Improve Clinical Outcomes? Am J Gastroenterol. 2016;111(1):87-92.

11. Bass NM, Mullen KD, Sanyal A, Poordad F, Neff G, Leevy CB, et al. Rifaximin treatment in hepatic encephalopathy. N Engl J Med. 2010;362(12):1071-81.

12. Wundke R, Altus R, Sandford J, Wigg A. Improving management of oesophageal varices in patients with cirrhosis. Qual Saf Health Care. 2010;19(6):536-41.

13. Kennedy NA, Rodgers A, Altus R, McCormick R, Wundke R, Wigg AJ. Optimisation of hepatocellular carcinoma surveillance in patients with viral hepatitis: a quality improvement study. Internal Medicine Journal. 2013;43(7):772-7.

14. Davila JA, Morgan RO, Richardson PA, Du XL, McGlynn KA, El-Serag HB. Use of surveillance for hepatocellular carcinoma among patients with cirrhosis in the United States. Hepatology. 2010;52(1):132-41.

15. Kanwal F, Kramer JR, Buchanan P, Asch SM, Assioun Y, Bacon BR, et al. The quality of care provided to patients with cirrhosis and ascites in the department of veterans affairs. Gastroenterology. 2012;143(1):70-7.

16. Volk ML, Tocco RS, Bazick J, Rakoski MO, Lok AS. Hospital readmissions among patients with decompensated cirrhosis. American Journal of Gastroenterology. 2012;107(2):247-52.

17. Bajaj JS, Reddy KR, Tandon P, Wong F, Kamath PS, Garcia-Tsao G, et al. The 3-month readmission rate remains unacceptably high in a large North American cohort of patients with cirrhosis. Hepatology. 2016;64(1):200-8.

18. Wagner EH, Austin BT, Von Korff M. Organizing care for patients with chronic illness. Milbank Q. 1996;74(4):511-44.

19. Scott IA. Chronic disease management: a primer for physicians. Internal Medicine Journal. 2008;38(6):427-37.

20. McAlister FA, Stewart S, Ferrua S, McMurray JJJV. Multidisciplinary strategies for the management of heart failure patients at high risk for admission: a systematic review of randomized trials. J Am Coll Cardiol. 2004;44(4):810-9.

21. Gonseth J, Guallar-Castillon P, Banegas JR, Rodriguez-Artalejo F. The effectiveness of disease management programmes in reducing hospital re-admission in older patients with heart failure: a systematic review and meta-analysis of published reports. Eur Heart J. 2004;25(18):1570-95.

22. Roccaforte R, Demers C, Baldassarre F, Teo KK, Yusuf S. Effectiveness of comprehensive disease management programmes in improving clinical outcomes in heart failure patients. A meta-analysis.[Erratum appears in Eur J Heart Fail. 2006 Mar;8(2):223-4]. Eur J Heart Fail. 2005;7(7):1133-44.

23. Hunt SA, Abraham WT, Chin MH, Feldman AM. ACC/AHA 2005 Guideline Update for the Diagnosis and Management of Chronic Heart Failure in the Adult: a report of the American College of Cardiology/American Heart Association Task Force on Practice Guidelines (Writing Committee to Update the 2001 Guidelines for the Evaluation and Management of Heart Failure): developed in collaboration with the American College of Chest Physicians and the International Society for Heart and Lung Transplantation: endorsed by the Heart Rhythm Society. Circulation. 2005;112(12):e154-235.

24. Adams SG, Smith PK, Allan PF, Anzueto A, Pugh JA, Cornell JE. Systematic review of the chronic care model in chronic obstructive pulmonary disease prevention and management. Arch Intern Med. 2007;167(6):551-61.

25. Volk ML, Piette JD, Singal AS, Lok AS. Chronic disease management for patients with cirrhosis. Gastroenterology. 2010;139(1):14-6.e1.

26. Wigg AJ, McCormick R, Wundke R, Woodman RJ. Coordinated care in cirrhosis; the need for further randomized controlled trials. J Hepatol. 2014;60(2):465-6.

27. Wigg AJ, McCormick R, Wundke R, Woodman RJ. Efficacy of a chronic disease management model for patients with chronic liver failure. Clin Gastroenterol Hepatol. 2013;11(7):850-8 e1-4.

28. Kanwal F. Coordinating care in patients with cirrhosis. Clinical Gastroenterology & Hepatology. 2013;11(7):859-61.

29. Mayorga CA, Singal AG. Can chronic disease management programs improve outcomes in patients with cirrhosis? Gastroenterology. 2013;145(5):1153-5.

30. Chin J-K, Kaambwa B, Wigg AJ. A chronic disease management model for chronic liver failure is cost effective: cost effectiveness analysis. Journal of Gastroenterology and Hepatology. 2016;31(Suppl.2):95.

31. Morando F, Maresio G, Piano S, Fasolato S, Cavallin M, Romano A, et al. How to improve care in outpatients with cirrhosis and ascites: a new model of care coordination by consultant hepatologists. Journal of Hepatology. 2013;59(2):257-64.

32. Tapper EB, Finkelstein D, Mittleman MA, Piatkowski G, Chang M, Lai M. A Quality Improvement Initiative Reduces 30-Day Rate of Readmission for Patients With Cirrhosis. Clin Gastroenterol Hepatol. 2016;14(5):753-9.

33. Weinberger M, Oddone EZ, Henderson WG. Does increased access to primary care reduce hospital readmissions? Veterans Affairs Cooperative Study Group on Primary Care and Hospital Readmission. New England Journal of Medicine. 1996;334(22):1441-7.

34. Harris AH, Hill SR, Chin G, Li JJ, Walkom E. The role of value for money in public insurance coverage decisions for drugs in Australia: a retrospective analysis 1994-2004. Med Decis Making. 2008;28(5):713-22.

35. Wigg AJ, McCormick R, Wundke R, Woodman RJ. Efficacy of a chronic disease management model for patients with chronic liver failure. Clinical Gastroenterology & Hepatology. 2013;11(7):850-8.e1-4.

36. Wigg AJ, Chinnaratha MA, Wundke R, Volk ML. A chronic disease management model for chronic liver failure. Hepatology. 2015;61(2):725-8.

37. Bodenheimer T, Wagner EH, Grumbach K. Improving primary care for patients with chronic illness: the chronic care model, Part 2. JAMA. 2002;288(15):1909-14.

38. Lemmens KMM, Nieboer AP, Huijsman R. A systematic review of integrated use of disease-management interventions in asthma and COPD. Respir Med. 2009;103(5):670-91.

39. Arora S, Thornton K, Murata G, Deming P, Kalishman S, Dion D, et al. Outcomes of treatment for hepatitis C virus infection by primary care providers. New England Journal of Medicine. 2011;364(23):2199-207.

40. Tapper EB, Halbert B, Mellinger J. Rates of and Reasons for Hospital Readmissions in Patients With Cirrhosis: A Multistate Population-based Cohort Study. Clin Gastroenterol Hepatol. 2016;14(8):1181-8 e2.

41. Khan A, Tansel A, White DL, Kayani WT, Bano S, Lindsay J, et al. Efficacy of Psychosocial Interventions in Inducing and Maintaining Alcohol Abstinence in Patients With Chronic Liver Disease: A Systematic Review. Clin Gastroenterol Hepatol. 2016;14(2):191-202 e1-4; quiz e20.

42. Kanwal F, Asch SM, Kramer JR, Cao Y, Asrani S, El-Serag HB. Early outpatient follow-up and 30-day outcomes in patients hospitalized with cirrhosis. Hepatology. 2016;64(2):569-81.

43. Younossi ZM, Guyatt G. Quality-of-life assessments and chronic liver disease. American Journal of Gastroenterology. 1998;93(7):1037-41.

44. Kanwal F, Kramer J, Asch SM, El-Serag H, Spiegel BMR, Edmundowicz S, et al. An explicit quality indicator set for measurement of quality of care in patients with cirrhosis. Clinical Gastroenterology & Hepatology. 2010;8(8):709-17.

45. Petkov J, Harvey P, Battersby M. The internal consistency and construct validity of the partners in health scale: validation of a patient rated chronic condition self-management measure. Quality of Life Research. 2010;19(7):1079-85.

46. Glick H, Briggs A, Polsky D. Quantifying stochastic uncertainty and presenting results of cost effectiveness analyses. Expert review of pharmacoeconomics and outcomes research. 2001;1:25-36.

47. Briggs A, O'Brien B, Blackhouse G. Thinking outside the box: recent advances in the analysis and presentation of uncertainty in cost-effectiveness studies. Annual Reviews Public Health. 2002;23:377-401.

48. Ge PS, Runyon BA. Care coordination for patients with cirrhosis: a "win-win" solution for patients, caregivers, providers, and healthcare expenditures. Journal of Hepatology. 2013;59(2):203-4.

# Appendices/Data Collection Tools

## Appendix A – Baseline Collection Tool

| ***BASELINE – to be completed for ALL patients*** | | | **Yes** | **No** |
| --- | --- | --- | --- | --- |
| **Ensure patient consent for ALFIE Nurse’s access to medical records is documented in patient notes (if not, please ask medical team member who gained verbal consent to document this before proceeding with below items) | | |  |  |
| 1 | Inclusion/exclusion criteria reviewed? (ensure patient eligible) | |  |  |
| 2 | Informed consent signed?  The Date PISCF was provided to the patient: ___ / ___ / _____  Date patient signed: ___ / ___ / _____  ICF version / date: ___ / ___ / _____  Has the patient been provided with a copy of the signed informed consent form? | |  |  |
| 3 | Date of Birth:  _____ /_____ /_____  DD / MMM / YYYY | |  |  |
| 4 | Gender  Male  Female | |  |  |
| 5 | Ethnicity: _________________________________ | |  |  |
| 6 | Post code of residence | |  |  |
| 7 | Primary Aetiology of Liver Disease:  Hepatitis C  Alcohol  Hepatitis B  Autoimmune disease | (Choose only one)  PBC  PSC  Haemochromatosis  NASH  Other _____________________ |  |  |
| 8 | When was cirrhosis diagnosed? | Month ________ Year __________ |  |  |
| 9 | Allocate Participant ID on Patient Identification Log  Site number:  LMH – 1 QEH – 2 SCGH – 3 RAH - 4  Then allocate patient number according to your log  (ie at LMH the first patient’s ID is 1-001, the second is 1-002 etc) | |  |  |
| 10 | Do they have a carer living at home with them? | |  |  |
| 11 | CLDQ completed by patient | |  |  |
| 12 | EQ-5D-5L completed by patient | |  |  |
| 13 | Cirrhosis Knowledge Assessment Questionnaire completed by patient | |  |  |
| 14 | PIH completed by patient | |  |  |
| 15 | ASK-12 completed by patient | |  |  |
| 16 | Resource use survey completed by patient | |  |  |
| 17 | MELD and Child Pugh Scores completed? (Use Child pugh and MELD page) | |  |  |
| 18 | Concomitant medications recorded? (use con meds page) | |  |  |
| 19 | Comorbidities recorded (see comorbidities page) | |  |  |
| 20 | Charlson Comorbidity Index completed | |  |  |
| 21 | Randomised? (see Randomisation page) | |  |  |

## Appendix B – Patient Identification Card (intervention only)

| **Participant Name:**  **Please Contact CLD Nurse:**  Phone Number (within hours): | **Research Trial – ALFIE**  This patient is participating in a study investigating a new way of managing patients with cirrhosis of the liver – Chronic Disease Management.  As part of this study this patient is receiving case management for their liver disease and their case coordinator would appreciate being informed of any admission to hospital or symptoms of concern related to the liver.  If you require information please call the number provided. |
| --- | --- |

## Appendix C – Ascites and Hepatic Encephalopathy Assessment

**Timepoint Assessment Completed:**  Baseline (intervention only) - Date: ___ / ___/ __

End of Trial - Date: ____ / ____/ _____

| ***ASCITES*** | | **Yes** | **No** |
| --- | --- | --- | --- |
| 1 | Does the patient have or ever had ascites?  If yes, add to care plan and continue with questions below.  If no, go to HE assessment. |  |  |
| 2 | If yes, is the patient on diuretics? (if yes, add to medication chart) |  |  |
| 3 | Action Plan given to patient and discussed |  |  |
| 4 | Weight chart discussed |  |  |
| 5 | Does patient have digital scales? (if no, add to care plan) |  |  |
| 6 | Any prior episode of spontaneous Bacterial Peritonitis (if yes, add to Care Plan) |  |  |
| 7 | Any evidence of prior total protein level in ascitic fluid ≤ 10g/L? |  |  |
| 8 | Is patient prescribed SBP prophylaxis? (If yes, add to medication list) |  |  |

Comments:

| ***HEPATIC ENCEPHALOPATHY*** | | **Yes** | **No** |
| --- | --- | --- | --- |
| 1 | Has patient ever had diagnosis of HE? (If yes, add to care plan and continue with below) If no – go to question 6. |  |  |
| 2 | Is patient on lactulose? (ensure on medication chart) |  |  |
| 3 | Is patient on rifaxamin? (ensure on medication chart) |  |  |
| 4 | Action plan for HE discussed |  |  |
| 5 | Does the patient drive? (if yes, ensure fitness to drive reviewed) |  |  |
| 6 | Number connection test done SCORE: ________________ seconds |  |  |
| 7 | EncephalApp assessment done SCORE: ___________________ |  |  |
| 8 | Does the patient have minimal HE? |  |  |

Comments:

## Appendix D – Osteoporosis Assessment

**Timepoint ASSESSMENT COMPLETED:**  Baseline - Date: ____ / ____/ _____

End of Trial - Date: ____ / ____/ _____

| ***Osteoporosis*** | | **Yes** | **No** |
| --- | --- | --- | --- |
| 1 | Has the patient had a Bone Density Scan (DEXA)? (if no, add to care plan to refer for screening) |  |  |
| 2 | What was the outcome of the most recent DEXA? (complete when DEXA done)  Normal  Osteopenia (add to care plan, confirm supplements started and add to medication chart)  Osteoporosis (add to care plan, confirm supplements and treatment started and add to medication chart) |  |  |
| 3 | Has patient had a vitamin D level taken? (if no add to care plan) |  |  |
| 4 | Outcome of last vitamin D level?  Normal  Mildly deficient (add to care plan, confirm supplements started and add to medication chart)  Severely deficient (add to care plan, confirm supplements started and add to medication chart) |  |  |
| 5 | If patient has osteoporosis/osteopenia are they on appropriate bone treatment? (ie vitamin D and calcium supplementation if osteopenic, Vitamin D and bisphosphonate if osteoporotic)  If No, please comment on why: |  |  |

Comments:

## Appendix E – Alcohol Assessment

**Timepoint ASSESSMENT COMPLETED:**  Baseline - Date: ____ / ____/ _____

End of Trial - Date: ____ / ____/ _____

| ***Alcohol use – prior to today’s assessment*** | | **Yes** | **No** |
| --- | --- | --- | --- |
| 1 | Has the patient drunk any alcohol in the last 6 months? (if yes, put on care plan) |  |  |
| 2 | If abstinent, date of last drink of alcohol (month and year) ______ / _______  month / year |  |  |
| 3 | If yes, specify alcohol amount:  Last 6 months:  Light (<2/day)  Moderate (2-4/day)  Heavy (>4/day)  Last 2 weeks:  Nil  Light (<2/day)  Moderate (2-4/day)  Heavy (>4/day) |  |  |
| 4 | Has patient seen Drug and Alcohol Services? (if no, arrange during inpatient stay) |  |  |
| 5 | Has an anticraving agent been prescribed? (If yes, add to medication list) |  |  |
| 6 | Is an anticraving agent being taken? (check with patient) |  |  |
| 7 | Has patient been referred to a psychologist for CBT/counselling? ( If no, add to plan of care for MHCP with GP and referral to psychologist) |  |  |

Comments:

## Appendix F – Variceal Management Assessment

**Timepoint ASSESSMENT COMPLETED:**  Baseline - Date: ____ / ____/ _____

End of Trial - Date: ____ / ____/ _____

| ***VARICES*** | | **Yes** | **No** |
| --- | --- | --- | --- |
| 1 | Has the patient had an Endoscopy? (if no, add to care plan to refer for screening) |  |  |
| 2 | Has the patient ever had a variceal bleed? (If yes, add to care plan) |  |  |
| 3 | Confirm education given regarding variceal bleeding cause and prevention |  |  |
| 4 | Assessment of eligibility for Baveno VI Criteria  Fibroscan < 25kPa – Date fibroscan done __ / __ / ____  Platelets > 110 – Platelet level ______ Date __ / __ / ____  If fibroscan not done, ensure this is on the care plan |  |  |
| 5 | What was the date and outcome of the most recent endoscopy?  Date: ____ / ____/ _____  No varices  Small varices (<5mm, or Grade 1 or 2)  Large varices/High risk varices (>5mm, or Grade 3)  No endoscopy done  Endoscopy not required due to Baveno VI criteria |  |  |
| 6 | According to the outcome above and the protocol for variceal screening and surveillance, what is the plan of care? (ensure add to care plan and recalls)  Baveno VI criteria – fibroscan due ____ / ____ /_______  Endoscopy in 3 years due ____ / ____ /_______  Endoscopy in 2 years due ____ / ____ /_______  Endoscopy in 1 year due ____ / ____ /_______  Variceal Ligation (eradication) ____ / ____ /_______  TIPS done ____ / ____ /_______  Primary prophylaxis (please add to medication chart and care plan)  Secondary prophylaxis post variceal bleed (please add to medication chart and care plan) |  |  |
| 7 | Was the patient adherent to variceal bleeding prevention protocols (answer only at end of trial) |  |  |
| 8 | Prophylaxis – primary or secondary  What is the patient’s pulse rate? ____________ bpm  (ensure that the patient is resting comfortably for 10 minutes prior to taking pulse rate) |  |  |

Comments:

## Appendix G – Nutrition Assessment

**Timepoint ASSESSMENT COMPLETED:**  Baseline - Date: ____ / ____/ _____

End of Trial - Date: ____ / ____/ _____

| ***Nutrition*** | | **Yes** | **No** |
| --- | --- | --- | --- |
| 1 | Has patient seen a dietitian about their liver disease? |  |  |
| 2 | Does patient have:  Obvious malnutrition  Concomitant diabetes  Refractory ascites  Struggling/not understanding diet or restrictions  Then dietitian referral is required. Date referred: ___ / ___ / _____ |  |  |
| 3 | Has the nutrition action plan been discussed with the patient? |  |  |

## Appendix H – Carer and GP Assessment

Post randomisation (intervention only) - Date: ___ / ___/ __

| ***CARER*** | | **Yes** | **No** |
| --- | --- | --- | --- |
| 1 | Does the patient have a carer? |  |  |
| 2 | If yes, does the carer have any concerns/issues regarding the patient’s liver disease? |  |  |
| 3 | Ensure that if carer living with the patient that the carer is educated using the liver disease booklet. |  |  |
| 4 | Does Carer need more support or information about accessing support?  If Yes – describe: |  |  |

Comments: _______________________________________________________________

| ***General Practitioner*** | | **Yes** | **No** |
| --- | --- | --- | --- |
| 1 | Does the patient have a regular GP? If no, enter this on the care plan and ensure patient gets access to regular GP/regular GP Practice. |  |  |
| 2 | Are GP Details documented? If No, ensure details documented |  |  |
| 3 | Indicate which of the following the patient needs:  GP Management Plan (good for most patients)  Team Care Arrangement (best for patients with multiple chronic diseases)  Mental Health Care Plan - (best for patients with alcohol dependence/abuse issues) patient will get money back from [Medicare](https://www.healthdirect.gov.au/medicare) for up to 10 individual or 10 group appointments with an [allied mental health service](https://www.healthdirect.gov.au/allied-health) in a year.  If any of these required, enter onto care plan and provide information required to GP |  |  |
| 4 | Call patient’s general practitioner informing them of patients involvement in chronic liver disease case management.  Record how the GP would like their updates about their patient:  Fax (record fax number) ______________________  Email (record email address) __________________________  Telephone (ensure right telephone number) ________________________ |  |  |

Comments:

## Appendix I – Home visit form

| Date | Date of Home Visit ____ /_____ /_____  DD / MMM / YYYY |
| --- | --- |
| Timing | Scheduled within 7 days of hospitalization  Rapid response for review of symptoms  Time at start _____ Time at end _______ total number of minutes ___________ |
| Rapid response? | If Rapid response, what is the symptom for review:  _________________________________________________________________  N/A - scheduled |
| Education Booklet | Confirm that patient still has education booklet (if not provide another one)  Continue education – particularly regarding symptoms the patient may not have experienced yet |
| S&S | Assess for other signs of complications for liver disease not previously diagnosed or may have been diagnosed since seeing the patient last.  Consider assessment for mild HE – NCT and Encephalapp |
| Medication Management | Discuss medication management – look at the patient’s answers to the ask12 medication survey and come up with strategies.  Would a webster pack assist adherence? Y  N  (if Yes, add to care plan and arrange)  Ensure that medications prescribed are available to patient  Check the medicine cupboard and around the home. Are there out of date medications? Are they doubling up on some medications, not taking medications, confused about what to take when? Do they need a home medicines review from a pharmacist? |
| Carer support | Consider the needs of a person’s carer. Can they continue to provide care and support? Is there a backup person/plan if the main carer becomes ill or needs some respite/holidays?  Ensure the carer knows about the booklet and encourage them to read it.  Carer is aware of liver nurse phone number and when to call |
| Care Plan | Please see overleaf some other issues you may wish to consider  Ensure all new issues and interventions are added to Care Plan  Ensure patient aware of specialist appointment date |

Study Coordinator (print) : ___________________ Signature: ________________________ Other home visit considerations:

- Assess self-management, anxiety, depression and lifestyle risks
- What are the health problems of greatest importance to the patient?
- What about their tobacco and alcohol use, sleep patterns, physical activity and nutrition?
- Consider psychosocial factors such as their perception of loneliness, bereavement or loss of motivation.
- What are their social needs and the extent and availability of social support? (including family, carers, neighbours and friends)
- Ask questions about hobbies, pets and activities to understand if the patient is socially isolated?
- Consider mobility and balance, including walking, transfers and climbing stairs. Are they currently in pain? Consider use of a pain score.
- maintaining personal hygiene, including bathing, grooming, toileting, continence and dressing
- eating and drinking. Are daily activities (chores, meal preparation, shopping) a problem?
- What transport do they use? Do they shop, prepare meals? How do they manage with home maintenance and housekeeping?
- Consider the abilities and limitations within the person’s living environment including safety issues, which may require resolution (ie slip/trip hazards, equipment, personal alarm)
- Is transport assistance required? Does the patient need assistance with making and attending appointments with specialists/allied health, etc

## Appendix J – Hospital Admission Log

Date of randomisation: ___ / ___ / ____

**Period of review (tick only one):**

□ Randomisation Date ___ / ___ / ____ to 12 months ___ / ___ / ____

□ 12 months ___ / ___ / ____to trial end ___ / ___ / ____

( NB If currently in hospital this counts as an admission in the time period being considered)

| Admission 1 |
| --- |
| Admission Date ___ / ___ / ____ Medically Stable for D/C Date ___ / ___ / ____  Discharge Date ___ / ___ / ____  Primary reason for admission as per D/C summary: __________________________  Liver Related Yes  No  Was this a decompensation Event? Yes  No  Emergency  or Elective  (Tick only one)  Number of days in ICCU (please state ‘0’ if not in ICCU): _______ |
| Admission 2 |
| Admission Date ___ / ___ / ____ Medically Stable for D/C Date ___ / ___ / ____  Discharge Date ___ / ___ / ____  Primary reason for admission as per D/C summary: __________________________  Liver Related Yes  No  Was this a decompensation Event? Yes  No  Emergency  or Elective  (Tick only one)  Number of days in ICCU (please state ‘0’ if not in ICCU): _______ |
| Admission 3 |
| Admission Date ___ / ___ / ____ Medically Stable for D/C Date ___ / ___ / ____  Discharge Date ___ / ___ / ____  Primary reason for admission as per D/C summary: __________________________  Liver Related Yes  No  Was this a decompensation Event? Yes  No  Emergency  or Elective  (Tick only one)  Number of days in ICCU (please state ‘0’ if not in ICCU): _______ |
| Admission 4 |
| Admission Date ___ / ___ / ____ Medically Stable for D/C Date ___ / ___ / ____  Discharge Date ___ / ___ / ____  Primary reason for admission as per D/C summary: __________________________  Liver Related Yes  No  Was this a decompensation Event? Yes  No  Emergency  or Elective  (Tick only one)  Number of days in ICCU (please state ‘0’ if not in ICCU): _______ |

| Admission 5 |
| --- |
| Admission Date ___ / ___ / ____ Medically Stable for D/C Date ___ / ___ / ____  Discharge Date ___ / ___ / ____  Primary reason for admission as per D/C summary: __________________________  Liver Related Yes  No  Was this a decompensation Event? Yes  No  Emergency  or Elective  (Tick only one)  Number of days in ICCU (please state ‘0’ if not in ICCU): _______ |
| Admission 6 |
| Admission Date ___ / ___ / ____ Medically Stable for D/C Date ___ / ___ / ____  Discharge Date ___ / ___ / ____  Primary reason for admission as per D/C summary: __________________________  Liver Related Yes  No  Was this a decompensation Event? Yes  No  Emergency  or Elective  (Tick only one)  Number of days in ICCU (please state ‘0’ if not in ICCU): _______ |
| Admission 7 |
| Admission Date ___ / ___ / ____ Medically Stable for D/C Date ___ / ___ / ____  Discharge Date ___ / ___ / ____  Primary reason for admission as per D/C summary: __________________________  Liver Related Yes  No  Was this a decompensation Event? Yes  No  Emergency  or Elective  (Tick only one)  Number of days in ICCU (please state ‘0’ if not in ICCU): _______ |
| Admission 8 |
| Admission Date ___ / ___ / ____ Medically Stable for D/C Date ___ / ___ / ____  Discharge Date ___ / ___ / ____  Primary reason for admission as per D/C summary: __________________________  Liver Related Yes  No  Was this a decompensation Event? Yes  No  Emergency  or Elective  (Tick only one)  Number of days in ICCU (please state ‘0’ if not in ICCU): _______ |
| Admission 9 |
| Admission Date ___ / ___ / ____ Medically Stable for D/C Date ___ / ___ / ____  Discharge Date ___ / ___ / ____  Primary reason for admission as per D/C summary: __________________________  Liver Related Yes  No  Was this a decompensation Event? Yes  No  Emergency  or Elective  (Tick only one)  Number of days in ICCU (please state ‘0’ if not in ICCU): _______ |

## Appendix K – Outpatient Attendance Log

Date of randomisation: ___ / ___ / ____

Period of review (tick only one):

Randomisation Date ___ / ___ / ____ to 12 months ___ / ___ / ____

12 months ___ / ___ / ____to trial end ___ / ___ / ____

| Outpatient review 1 |
| --- |
| Appointment Date ___ / ___ / ____  Attended? Yes  No  If no Why? Did not turn up  Cancelled |
| Outpatient review 2 |
| Appointment Date ___ / ___ / ____  Attended? Yes  No  If no Why? Did not turn up  Cancelled |
| Outpatient review 3 |
| Appointment Date ___ / ___ / ____  Attended? Yes  No  If no Why? Did not turn up  Cancelled |
| Outpatient review 4 |
| Appointment Date ___ / ___ / ____  Attended? Yes  No  If no Why? Did not turn up  Cancelled |
| Outpatient review 5 |
| Appointment Date ___ / ___ / ____  Attended? Yes  No  If no Why? Did not turn up  Cancelled |
| Outpatient review 6 |
| Appointment Date ___ / ___ / ____  Attended? Yes  No  If no Why? Did not turn up  Cancelled |

| Outpatient review 7 |
| --- |
| Appointment Date ___ / ___ / ____  Attended? Yes  No  If no Why? Did not turn up  Cancelled |
| Outpatient review 8 |
| Appointment Date ___ / ___ / ____  Attended? Yes  No  If no Why? Did not turn up  Cancelled |
| Outpatient review 9 |
| Appointment Date ___ / ___ / ____  Attended? Yes  No  If no Why? Did not turn up  Cancelled |
| Outpatient review 10 |
| Appointment Date ___ / ___ / ____  Attended? Yes  No  If no Why? Did not turn up  Cancelled |
| Outpatient review 11 |
| Appointment Date ___ / ___ / ____  Attended? Yes  No  If no Why? Did not turn up  Cancelled |
| Outpatient review 12 |
| Appointment Date ___ / ___ / ____  Attended? Yes  No  If no Why? Did not turn up  Cancelled |
| Outpatient review 13 |
| Appointment Date ___ / ___ / ____  Attended? Yes  No  If no Why? Did not turn up  Cancelled |

If more than 13 appointments in 12 months, please print this page a second time.

## Appendix L – West Haven Criteria

**Date of assessment ____ / ____ / ________**

| **West Haven Criteria** | |
| --- | --- |
| Grade | Criteria |
| 1 | Trivial lack of awareness |
|  | Euphoria or anxiety |
|  | Shortened attention |
|  | Impaired performance of addition |
| 2 | Lethargy or apathy |
|  | Minimal disorientation of time or place |
|  | Subtle personality changes |
|  | Inappropriate behaviour |
|  | Lethargy or apathy |
| 3 | Somnolence to semi-stupor but responsibe to verbal stimuli |
|  | Confusion |
|  | Gross disorientation |
| 4 | Coma (unresponsive to verbal or noxious stimuli) |

Principal Investigator : ______________________________

Signature: ______________________________________

Date: _________________________

## Appendix M – Contact Log

| **Nurse Contact Log** | **ALFIE** | Participant  ID | Participant Initials |
| --- | --- | --- | --- |
|  |  | __- __ __ __ | __ __ __ |

This is completed for all Intervention patients to document all contacts with the patient or their GP.

| **Contact type** | **Date** | **SIGNED** |
| --- | --- | --- |
| Scheduled Home Visit  Unscheduled Home visit (rapid response)  Scheduled telephone contact  Unscheduled telephone contact  GP Fax/ Email / Telephone call (please circle which one) | ____/______/___  DD/MMM/YY | ____________ |
| Scheduled Home Visit  Unscheduled Home visit (rapid response)  Scheduled telephone contact  Unscheduled telephone contact  GP Fax/ Email / Telephone call (please circle which one) | ____/______/___  DD/MMM/YY | ____________ |
| Scheduled Home Visit  Unscheduled Home visit (rapid response)  Scheduled telephone contact  Unscheduled telephone contact  GP Fax/ Email / Telephone call (please circle which one) | ____/______/___  DD/MMM/YY | ____________ |
| Scheduled Home Visit  Unscheduled Home visit (rapid response)  Scheduled telephone contact  Unscheduled telephone contact  GP Fax/ Email / Telephone call (please circle which one) | ____/______/___  DD/MMM/YY | ____________ |
| Scheduled Home Visit  Unscheduled Home visit (rapid response)  Scheduled telephone contact  Unscheduled telephone contact  GP Fax/ Email / Telephone call (please circle which one) | ____/______/___  DD/MMM/YY | ____________ |
| Scheduled Home Visit  Unscheduled Home visit (rapid response)  Scheduled telephone contact  Unscheduled telephone contact  GP Fax/ Email / Telephone call (please circle which one) | ____/______/___  DD/MMM/YY | ____________ |
| Scheduled Home Visit  Unscheduled Home visit (rapid response)  Scheduled telephone contact  Unscheduled telephone contact  GP Fax/ Email / Telephone call (please circle which one) | ____/______/___  DD/MMM/YY | ____________ |

Print more as required.

## Appendix N– Child Pugh and MELD Score

**Timepoint ASSESSMENT COMPLETED:**  Baseline - Date: ____ / ____/ _____

3 month - Date: ____ / ____/ _____

6 month - Date: ____ / ____/ _____

Intervention only

12 month - Date: ____ / ____/ _____

18 month - Date: ____ / ____/ _____

End of Trial - Date: ____ / ____/ _____

| CHILDS PUGH SCORE – go to [**https://www.mdcalc.com/child-pugh-score-cirrhosis-mortality**](https://www.mdcalc.com/child-pugh-score-cirrhosis-mortality) | |
| --- | --- |
| Blood test date: ____________________ (most recent – closest to date due)  Bilirubin: ______  Albumin: ______  INR: __________  **Ascites**  None  Slight/controlled on treatment  Moderate | **HE**  None  Grade 1-2  Grade 3-4  **RESULT**  A5  A6  B7  B8  B9  C10  C11  C12 |
| MELD- go to [**https://www.mdcalc.com/meld-score-model-end-stage-liver-disease-12-older**](https://www.mdcalc.com/meld-score-model-end-stage-liver-disease-12-older) | |
| Dialysis ≥ 2 times in the past week?  No  Yes  From Blood test date: ______________________  Creatinine: ___________  MELD _______________  Bilirubin: _____________  INR: ________________  Na+: ________________ | |

Comments:

## Appendix O – Co-morbidities

This is kept updated thoughout the trial but particularly at Baseline and end of study

| **Condition/Abnormality** | **Start Date** | **Stop DatE / Ongoing** | | |
| --- | --- | --- | --- | --- |
|  | ____/______/____  DD/MMM/YY | ____/______/____  DD/MMM/YY | OR |  |
|  | ____/______/____  DD/MMM/YY | ____/______/____  DD/MMM/YY | OR |  |
|  | ____/______/____  DD/MMM/YY | ____/______/____  DD/MMM/YY | OR |  |
|  | ____/______/____  DD/MMM/YY | ____/______/____  DD/MMM/YY | OR |  |
|  | ____/______/____  DD/MMM/YY | ____/______/____  DD/MMM/YY | OR |  |
|  | ____/______/____  DD/MMM/YY | ____/______/____  DD/MMM/YY | OR |  |
|  | ____/______/____  DD/MMM/YY | ____/______/____  DD/MMM/YY | OR |  |
|  | ____/______/______  DD/MMM/YY | ____/______/____  DD/MMM/YY | OR |  |
|  | ____/______/____  DD/MMM/YY | ____/______/____  DD/MMM/YY | OR |  |
|  | ____/______/____  DD/MMM/YY | ____/______/____  DD/MMM/YY | OR |  |
|  | ____/______/____  DD/MMM/YY | ____/______/____  DD/MMM/YY | OR |  |

Comments:

Study Coordinator : ______________________________

Signature: ______________________________________

## Appendix P – Concomitant Medications

From the ASK 12 Questionnaire –

Were there any issues identified in the forgetfulness or behavior sections that suggest that a webster/blister pack would be useful?  Yes  No

If yes, please add medication adherence as an issue on the care plan

| **CM #** | **Medication / Drug Name**  **(generic name preferred)** | **Start Date** | **Stop Date / Ongoing** | **Indication** | **Dose**  **(units)** | **Route*** | **Frequency**** |
| --- | --- | --- | --- | --- | --- | --- | --- |
|  |  | **____/_______/_______**  **DD / MMM / YYYY** | **____/_______/_______**  **DD / MMM / YYYY**  **Ongoing** |  |  |  |  |
|  |  | **____/_______/_______**  **DD / MMM / YYYY** | **____/_______/_______**  **DD / MMM / YYYY**  **Ongoing** |  |  |  |  |
|  |  | **____/_______/_______**  **DD / MMM / YYYY** | **____/_______/_______**  **DD / MMM / YYYY**  **Ongoing** |  |  |  |  |
|  |  | **____/_______/_______**  **DD / MMM / YYYY** | **____/_______/_______**  **DD / MMM / YYYY**  **Ongoing** |  |  |  |  |
|  |  | **____/_______/_______**  **DD / MMM / YYYY** | **____/_______/_______**  **DD / MMM / YYYY**  **Ongoing** |  |  |  |  |

***ROUTE:**  ****FREQUENCY:**

IV (intravenous) SC (subcutaneous) QD: once daily BID: twice a day

PR (per rectum) INH (inhaled) TID: three times a day QID: four times a day

IM (intramuscular) TD (transdermal) PRN: as occasion requires single dose

SL (sublingual) TOP (topic al) Every morning Every evening

PO (per os – by mouth) Every other hour Other, specify

Others, specify-----------

## Appendix Q – End of Study Assessment Form

**To be completed on all patients (Intervention AND Control)**

| **Outcomes** | | **Yes** | **No** |
| --- | --- | --- | --- |
| 1 | Patient has **completed** the study (if yes complete table below)  Date of End of Study ____ / ____ / ______ |  |  |
| 2 | Patient has **DIED**  Date of Death ____ / ____ / ______ |  |  |
| 3 | Patient has been placed on the **transplant waiting list** (Complete below when placed on list)  Date of placement on waiting list ____ / ____ / ______ |  |  |
| 4 | Patient has been **LOST** to follow up  Date of last contact ____ / ____ / ______ |  |  |
| 5 | Date of Palliative Care Referral ____ / ____ / ______ |  |  |

**If patient completed the study please ensure the following:**

| ***End of Study Activities*** | | **Yes** | **No** |
| --- | --- | --- | --- |
| 1 | CLDQ completed by patient |  |  |
| 2 | EQ5D 5L completed by patient |  |  |
| 3 | Cirrhosis Knowledge Assessment Questionnaire completed by patient |  |  |
| 4 | PIH completed by patient |  |  |
| 5 | ASK-12 completed by patient |  |  |
| 6 | Resource Use Questionnaire completed by patient |  |  |
| 7 | Patient satisfaction survey completed by patient |  |  |
| 8 | MELD and Child Pugh Scores completed? |  |  |
| 9 | Concomitant medications recorded? |  |  |
| 10 | Hospital Admissions form completed? |  |  |
| 11 | Outpatient appointments form completed? |  |  |
| 12 | Nursing Contacts page completed? |  |  |
| 13 | All assessment forms completed (Appendix C, D, E, F, G, R) |  |  |

Study Coordinator : ______________________________

Signature: ______________________________________

Date: _________________________

## Appendix R – HCC Assessment form

**Timepoint Ax COMPLETED:**  Baseline (intervention only) - Date: ____ / ____/ _____

End of Trial - Date: ____ / ____/ _____

| ***HCC*** | | **Yes** | **No** |
| --- | --- | --- | --- |
| 1 | Has the patient had an upper abdominal ultrasound? (if no, add to care plan to refer for screening) |  |  |
| 2 | How many ultrasounds has the patient had the last 2 years  0  1 Date : ____ / ____/ _____  2 Date : ____ / ____/ _____  3 Date : ____ / ____/ _____  4 Date : ____ / ____/ _____  N/A – patient only just diagnosed with cirrhosis (baseline only option) |  |  |
| 3 | Was HCC found in any of the above ultrasounds? |  |  |

Comments:

## Appendix S – Care Plan

**ALFIE TRIAL CARE PLAN**

| Date: | Date of randomization: ___ / ___ / _____ | |  |
| --- | --- | --- | --- |
| Aetiology : | Childs Pugh: MELD: | Consultant: | |
| **Active Problem list:** | **Actions / Plan** | | **Date resolved** |
| **#** |  | |  |
| **#** |  | |  |
| **#** |  | |  |
| **#** |  | |  |
| **#** |  | |  |
| **#** |  | |  |
| **#** |  | |  |
| **#** |  | |  |
| **#** |  | |  |
| **#** |  | |  |
| **#** |  | |  |
| **Comment/ Consultant instructions:**  **Signature: ……………………………………………………..Date:…………………………………** | | | |

Not for filing in MR

ALFIE TRIAL

**CARE PLAN REVIEW DATES**

Please document when the care plan was discussed/reviewed with the treating Specialist and ask the Specialist to sign below:

Review Date: ____________ Signed: _______________________ Name: ___________________________

Review Date: ____________ Signed: _______________________ Name: ___________________________

Review Date: ____________ Signed: _______________________ Name: ___________________________

Review Date: ____________ Signed: _______________________ Name: ___________________________

Review Date: ____________ Signed: _______________________ Name: ___________________________

Review Date: ____________ Signed: _______________________ Name: ___________________________

Review Date: ____________ Signed: _______________________ Name: ___________________________

Review Date: ____________ Signed: _______________________ Name: ___________________________

Review Date: ____________ Signed: _______________________ Name: ___________________________

Review Date: ____________ Signed: _______________________ Name: ___________________________

Review Date: ____________ Signed: _______________________ Name: ___________________________

Review Date: ____________ Signed: _______________________ Name: ___________________________

Review Date: ____________ Signed: _______________________ Name: ___________________________

Review Date: ____________ Signed: _______________________ Name: ___________________________

Review Date: ____________ Signed: _______________________ Name: ___________________________

Review Date: ____________ Signed: _______________________ Name: ___________________________

Review Date: ____________ Signed: _______________________ Name: ___________________________

Review Date: ____________ Signed: _______________________ Name: ___________________________

## Appendix T – Charlson Comorbidity index

| **Charlson Comorbidity Index** | **ALFIE** | **Subject**  **ID** | **Subject Initials** |
| --- | --- | --- | --- |
|  |  | __- __ __ __ | __ __ __ |

1. **Has the patient had a myocardial infarction? (MI)**

- Yes
- No

*Criteria: Myocardial infarction includes patients with one or more definite or probably myocardial infarction. These patients should have been hospitalised for chest pain or an equivalent clinical event and have electrocardiographic and/or enzyme changes. Patients with electrocardiographic changes alone who have no clinical history are not designated as having an infarction.*

**2. Has the patient been hopsitalised or treated for heart failure? (CHF)**

- Yes
- No

*Criteria: Congestive heart failure includes patients who have had exertional or paroxysmal nocturnal dyspnoea and who have responded symptomatically (or on physical examination) to digitalis, diuretics, or afterload reducing agents. It does not include patients who are on one of those medications but who have had no response and no evidence of improvement of physical signs with treatment.*

**3. Does the patient have peripheral vascular disease? (PVD)**

- Yes
- No

*Criteria: Peripheral vascular includes patients with intermittent claudication or those who had a bypass for arterial insufficiency, those with gangrene or acute arterial insufficiency, and those with a treated or untreated thoracic or abdominal aneurysm (6cm or more).*

**4. Has the patient had a CVA or transient ischaemic disease? (CVA)**

- Yes
- No

*Criteria: Cerebrovascular disease includes patients with a history of a cerebrovascular accident with minor or no residual, and patient who have had transient ischaemic attacks. If the CVA resulted in hemiplegia, code only hemiplegia.*

**5. Does the patient have hemiplegia? (PLEGIA)**

- Yes
- No

*Criteria: This includes patients with a hemiplegia or paraplegia, whether it occureed as a result of a cerebrovascular accident or other condition.*

**6. Does the patient have asthma, chronic lung disease, chronic bronchitis or emphysema? (COPD)**

- Yes
- No

*Criteria: Pulmonary disease includes patients with asthma, chronic bronchitis, emphysema, and other chronic lung disease who have ongoing symptoms such as dyspnoea or cough, with mild or moederate activity. This includes patients who are dyspnoeic with slight activity, with or without treatment and those who are dyspnoeic with moderate activity despite treatment, as well as patients who are dyspnoeic at rest, despite treatment, those who require constant oxygen, those with CO2 retention and those with a baseline PO2 below 50 torr.*

**7. Does the patient have diabetes that requires treatment? (DM)**

- Yes
- No

*Criteria: Diabetes includes all patients with diabetes treated with insulin or oral hypoglycaemic, but not diet alone. Diabetes during treatment is not counted.*

**7a. Does the patient have end organ damage from diabetes? (DM ENDORGAN)**

- Yes
- No

*Criteria: This includes patients with retinopathy, neuropathy, or nephropathy attributable to diabetes.*

**8. Does the patient have moderate or severe renal disease? (RENAL)**

- Yes
- No

*Criteria: Moderate renal insufficiency includes patients with a serum creatinine >3mg/dl. Severe renal disease includes patients on dialysis, those who had a transplant, and those with uremia.*

**9. Does the patient have a chronic liver disease (MILDLIVER)**

- Yes
- No

*Criteria: Mild liver disease consists of chronic hepatitis (B or C) or cirrhosis without portal hypertension*

**9a. Does the patient have moderate to severe liver disease? (SEVERELIVER)**

- Yes
- No

*Criteria: Moderate liver disease consists of cirrhosis with portal hypertension but without bleeding. Severe liver disease consists of patients with ascites, chronic jaundice, portal hypertensions or a history of variceal bleeding or those who have had liver transplant.*

**10. Has the patient had gastric or peptic ulcers? (ULCERS)**

- Yes
- No

*Criteria: Peptic ulcer disease includes patient who have required treatment for ulcer disease, including those who have bled from ulcers.*

**11. Has the patient had cancer (other than basal cell skin cancer)? (CANCER)**

- Yes
- No

If yes, which:

- - Lymphoma?
  - Leukaemia?
  - Solid tumor (which?) ___________________

*Criteria: Lymphoma includes patients with Hedgkins, lymphosarcoma, Waldenstrom’s macroglobulinaemia, myeloma, and other lymphomas. Leukaemia includes patients with acute and chronic myelogenous leukemia, acute and chronic lymphocytic leukaemia, and polycythaemia vera. Solid tumour consists of patients with solid tumours without documented metastases, including breast, colon, lung, prostate, and a variety of other tumours.*

**11a. Has the patient had a metastatic solid tumour? (METASTASES)**

- Breast □ Lung
- Colon □ Prostate
- Melanoma
- Other _______________________

*Criteria: Metastatic cancer includes patients with metastatic solid tumours, including breast, lung, colon and other tumours.*

**12. Does the patient have Alzheimer’s, dementia from any aetiology or any serious cognitive impairment? (DEMENTIA)**

- Yes
- No

*Criteria: Dementia includes patients with moderate to severe chronic cognitive deficit resulting in impaired function from any cause.*

**13. Does the patient have any rheumatic or connective tissue disease? (RHEUMATIC)**

- Yes
- No

*Criteria: Rheumatologic disease includes patients with systemic lupus erythematous, polymyositis, mixed connective tissue disease, rheumatoid arthritis, polymyositis, polymyalgia rheumatica, vasculitis, sarcoidosis, Sjogrens syndrome or any other systemic vasculitis.*

**14. Does the patient have HIV or AIDS? (HIV)**

- Yes
- No

*Criteria: Acquired immune deficiency syndrome includes patients with definite or probably AIDS i.e. AIDS related complex, and those who are HIV positive and asymptomatic.*

**15. Does the patient have hypertension? (HBP)**

- Yes
- No

*Criteria: Hypertension includes patients who have systolic pressures >140mmHg and/or diastolic pressures >90mmHg if without diabetes or renal disease, as well as controlled hypertensives; or patients with diabetes or renal disease who have systolic pressures >140mmHg or diastolic pressures >80mmHg.*

**16. Has the patient had decubitus ulcers, peripheral skin ulcers or repeated episodes of cellulitis?**

- Yes
- No

*Criteria: Partial thickness loss of skin over legs or back with open ulcers or two or more episodes of cellulitis requiring treatment with antibiotics, regardless of aetiology.*

**17. Does the patient have depression?**

- Yes
- No

*Criteria: Patients who are currently receiving treatment for depression, whether pharmacologic or psychotherapy, or cognitive behavioural therapy, or notes indicating that the patient has probably or definite depression.*

**18. Is the patient on warfarin or Coumadin?**

- Yes
- No

*Comments: ………………………………………………………………………………………………………………………………………………………..*

**…………………………………………………………………………………………………………………………………………………………………………**

## Appendix U – Diuretic Titration Guidelines for Trial Nurses

*This guideline has been developed to assist the ALFIE Nurses to provide timely diuretic titration to patients on these medications. As experienced RNs in the field of Gastroenterology and Hepatology, the ALFIE nurses have received training in diuretic titration during the Investigator Meetings in November 2017 and August 2018. Nurses are encouraged to check with a medical officer if in doubt.*

| Medicine Name: Frusemide (Furosemide) and Spironolactone (Adult) | |
| --- | --- |
| **Formulation:** | Frusemide (Furosemide): 20 mg tablet and 40 mg tablet  Spironolactone: 25mg tablet and 100mg tablet |
| **Specific Condition** | Non tense ascites secondary to advanced liver disease. |
| **Purpose and scope:** | Facilitate timely titration of diuretic treatment of non-tense ascites associated with liver failure, for adult patients enrolled in the ALFIE trial |
| **Limitations:** | Only applies to patients enrolled in the ALFIE trial |
| **Indications for use:** | Non-tense ascites associated with chronic liver disease:   - Patients must be able to monitor weight daily, either alone or with carer input. - Patients and/or carers must be able to understand and implement titration instructions. |
| **Contra-indications:** | - Patients with a known barrier to compliance. - Patients unable to be weighed daily. - Patients/carers unable to understand and implement titration instructions. - Patients with renal impairment who require a Medical Officers input regarding diuretic titration. - *Patients with stable chronic renal impairment may have their diuretics managed by the ALFIE nurse as per this guideline if instructed to do so by the patient’s attending Hepatologist.* - Patients with overt hepatic encephalopathy. |
| **Dose** | - This guideline covers patients who are currently prescribed frusemide (furosemide) and spironolactone by a medical officer for the management of non-tense ascites associated with chronic liver disease. - Doses of spironolactone and frusemide (furosemide) can be titrated up in increments of a half or full dose. - Usually both diuretics are titrated up together but at times dysfunction in electrolytes (Sodium or potassium) may necessitate titration of only one diuretic. - Doses must not exceed a maximum of frusemide (furosemide) 160mg daily or spironolactone 400mg daily. |
| **Frequency:** | ALFIE trial nurses may instruct the patient to increase their dose of frusemide (furosemide) or spironolactone no more than every 5 days (following receipt of electrolyte test results). |
| **Procedure for administration:** | Patients/carers will monitor weight daily and those failing to show adequate weight loss over a five day period and with normal kidney function and electrolytes will be directed by the ALFIE trial nurse to increase their dose of diuretics. |
| **Management of Complications:** | Complications of diuretics:  **Renal failure:** refer to treating medical officer  **Hepatic encephalopathy:** refer to treating medical officer  **Electrolyte disturbances:**  **Hypokalaemia:** reduce if K+ <hold frusemide (furosemide) if serum potassium <3 mmol/L and recheck bloods within 72hrs.  **Hyperkalaemia:** reduce Spironolactone if serum potassium >5.6 mmol/L. Hold spironolactone if serum potassium >6.0 mmol/L. Recheck bloods within 72hrs  **Hyponatraemia:** Reduced diuretics if sodium <130mmol/L. Hold diuretics if sodium < 125 mmol/L. Recheck bloods within 72hrs  **Muscle cramps:** Check electrolytes. If severe, may require discontinuation; Discuss with Medical Officer.  Complications are to be managed by the ALFIE trial nurse and/or treating Medical Officer depending on the severity. |
| **Monitoring requirements:** | - Diuretic titrations up in dose should be followed by a blood test in 5 days and electrolyte imbalance managed with consideration of concomitant therapy (conducted by either the ALFIE trial nurse and/or treating Medical Officer). - Diuretic titrations down in response to kidney or electrolyte dysfunction should be monitored closely with blood tests. |
| **Documentation:** | Any dose changes are to be documented in the patient’s outpatient medical record/EPAS and the GP informed. |

| REFERENCES | |
| --- | --- |
| This document has been developed based on the Southern Adelaide Local Health Network, Standing Medication Order for Frusemide (Furosemide) and Spironolactone (Adult): Issue Date 24/08/2017  Ascites [revised 2016 Mar]. In: eTG complete [Internet}. Melbourne: Therapeutic Guidelines Limited; 2017 Mar  European Association of the Sudy of the Liver EASL clinical practice guidelines on the management of ascites, spontaneous bacterial peritonitis, and hepatorenal syndrome in cirrhosis. J Hepatol. 2010;53 (3): 397 – 417.  Frusemide (2017) Retrieved July 5, 2017, from <http://www.amh.hcn.net.au>  Runyon BA. AASLD Practice Guideline: Management of Adult Patients with Ascites Due to Cirrhosis: update 2012. AASLD Pract. Guidel. 2012; 1-96.  Spironolactone (2017) Retrieved July 5, 2017, from http://www.amh.hcn.net.au. |  |

## Appendix V – ALFIE Interview Questions for Patients

Introduction

Hi, I’m XX. Thank you for your time today. I am not a member of the ALFIE trial but have been asked to assist the research team with conducting these phone interviews. The aim of this interview is to find out about your experience being part of the ALFIE trial.

Questions

1. Tell me about your experience of having your liver disease managed at <your hospital>.
2. Was anyone involved in your care besides doctors?
3. Did you have a special liver nurse in charge of your care?
4. If yes, could you tell us about your experience receiving care from this liver nurse?
5. Did you feel supported during your treatment?
6. Did you receive any education about your liver disease?
7. Did you learn about self-management for the complications of your liver disease?
8. Do you feel confident about receiving appropriate care for your liver disease?
9. What were some of the good things about the care you received for your liver disease?
10. Do you have any complaints about the care received?
11. Is there anything that would have made the care you received for your liver disease any better?
12. Do you have any other feedback about the care you received by the liver department at <your hospital>?

## Appendix W – ALFIE Interview Questions for Staff

Introduction

Hi, I’m XX. Thank you for your time today. I am not a member of the ALFIE trial but have been asked to assist the research team with conducting these phone interviews. The aim of this interview is to find out about your experience being part of the ALFIE trial.

Questions

1. Do you feel that the case management provided has helped the patients in the ALFIE intervention arm compared to standard of care?
2. What were the strengths/benefits of the intervention (case management)?
3. What were the weaknesses of the intervention (case management)?
4. What are the key issues that need to be discussed by the nurses in providing case management?
5. *(For Nurses only)*:
6. Did you feel confident to provide the intervention (case management)?
7. Were you adequately supported with the information provided (such as study protocols and information folders) to perform the intervention?
8. Did you have to be upskilled to provide this role?
9. Describe the learning opportunities given to you to enable you to fulfill this role.
10. Would you like to continue in this role after the trial ends? Why?
11. Would you like to learn more about caring for patients with cirrhosis of the liver?
12. What were the problems encountered in providing the intervention and how were they managed?
13. How can this model of care be improved further?
14. Do you have any other feedback about this model of care?
15. Would you like to see the role implemented at <your hospital> going forward?

Yes/No, please elaborate.
