# Supplementary material for: A qualitative enquiry into participants’ and practitioners’ experiences in the Australian Liver FaIlurE trial
Source: BMJ Open. 2025 Feb 16;15(2):e089666. doi: 10.1136/bmjopen-2024-089666 (PMC11831275; doi:10.1136/bmjopen-2024-089666)
Supplement: online supplemental table 1 [file bmjopen-15-2-s002.docx]

**Supplementary Table 1. ALFIE Qualitative Questions – Telephone interview guide for patients**

| **Introduction** | Hi, I’m XX. Thank you for your time today. I am not a member of the ALFIE trial but have been asked to assist the research team with conducting these phone interviews. The aim of this interview is to find out about your experience of being part of the ALFIE trial. |
| --- | --- |
| **Questions** |  |
| 1 | Tell me about your experience of having your liver disease managed at <your hospital>. |
| 2 | Was anyone involved in your care besides doctors? |
| 3 | Did you have a special liver nurse in charge of your care? |
|  | 1. If yes, could you tell us about your experience receiving care from this liver nurse? |
| 4 | Did you feel supported during your treatment? |
| 5 | Did you receive any education about your liver disease? |
| 6 | Did you learn about self-management for the complications of your liver disease? |
| 7 | What else did you learn about your liver disease? |
| 8 | Do you feel confident about receiving appropriate care for your liver disease? |
| 9 | What were some of the good things about the care you received for your liver disease? |
| 10 | Do you have any complaints about the care received? |
| 11 | Is there anything that would have made the care you received for your liver disease any better? |
| 12 | What are your expectations going forward regarding the care of your liver disease? |
| 13 | Do you have any other feedback about the care you received by the liver department at <your hospital>? |

**Supplementary Table 2. ALFIE Qualitative Questions – Telephone interview guide for nurses and doctors**

| **Introduction** | Hi, I’m XX. Thank you for your time today. I am not a member of the ALFIE trial but have been asked to assist the research team with conducting these phone interviews. The aim of this interview is to find out about your experience being part of the ALFIE trial. |
| --- | --- |
| **Questions** |  |
| 1 | Do you feel that the case management provided has helped the patients in the ALFIE intervention arm compared to the standard of care? |
| 2 | What were the strengths/benefits of the intervention (case management)? |
| 3 | What were the weaknesses of the intervention (case management)? |
| 4 | What are the key issues that need to be discussed by the nurses in providing case management? |
| *For Nurses only* |  |
| a | Did you feel confident to provide the intervention (case management)? |
| b | Did you have to be upskilled to provide this role? |
| c | Were you adequately supported with the training provided (such as study protocols and information folders) to perform the intervention? |
| d | Describe the learning opportunities given to you to enable you to fulfil this role. |
| e | Would you like to continue in this role after the trial ends? Why? |
| f | Are there any learning gaps that became apparent for you as part of participating in delivery of the trial intervention? |
| 5 | What were the problems encountered in providing the intervention and how were they managed? |
| 6 | How can this model of care be improved further? |
| 7 | Do you have any other feedback about this model of care? |
| 8 | Would you like to see the role implemented at <your hospital> going forward? |
|  | Yes/No, please elaborate. |
