# Supplementary material for: A qualitative enquiry into participants’ and practitioners’ experiences in the Australian Liver FaIlurE trial
Source: BMJ Open. 2025 Feb 16;15(2):e089666. doi: 10.1136/bmjopen-2024-089666 (PMC11831275; doi:10.1136/bmjopen-2024-089666)
Supplement: Supplementary file 3 [file bmjopen-2024-089666.reviewer_comments.pdf]

PEER REVIEW HISTORY

BMJ Open publishes all reviews undertaken for accepted manuscripts. Reviewers are asked to complete a checklist review form and are provided with free text boxes to elaborate on their assessment. These free text comments are reproduced below.

ARTICLE DETAILS

Title (Provisional)

A qualitative enquiry into participants' and practitioners' experiences in the Australian Liver FaIlurE trial

Authors

Ramachandran, Jeyamani; Pati, Anuradha; Wigg, Luisa; Narayana, Sumudu K; Lawn, Sharon; Muller, Kate; wigg, Alan

---

VERSION 1 - REVIEW

**Reviewer**	**1**
**Name**	**Harney, Brendan**
**Affiliation**	**Burnet Institute**
**Date**	**23-Jul-2024**
**COI**	**None**

Overall this is a well written paper providing a qualitative evaluation of a RCT on nurse-coordinated care to reduce liver-related emergency admissions. My comments are mostly minor in nature.

Methods

1. I understand you wanted to, and did, interview intervention participants and controls, as well as nurses and hep/gastro specialists, but purposive sampling is rather vague and should be elaborated on more. While written with implementation research as a focus, Palinkas et al 2015 (https://www.ncbi.nlm.nih.gov/pmc/articles/PMC4012002/ for example lists a wide variety of purposeful sampling strategies – does yours fall under this, or something else? As written, it seems to be more just a convenience sample of the pre-selected sub-groups. This is fine, but it just needs to be made clearer.

2. While it is stated that the interviewers were blinded to the interventional status, would "unblinding" not have occurred during the interviews given participants themselves would have known whether they were in the intervention or control group given it was not possible to be blinded? I think this should be acknowledged and discussed.

3. It is not clear when the interviews took place, were they during or once the intervention had completely finished? Please add this detail.

Results

4. Can you clarify, in table 1, where it does not say “control participants”, are these all quotes from the intervention participants. I assume so, but can you make this clearer by labelling in the same way?

Discussion

5. Your mentioned addiction and mental health in the discussion, were these common among study participants?. I cannot access the main paper via my institute, so can you please provide some brief details on this for the participants where you talk about alcohol being the primary aetiology for cirrhosis?

**Reviewer**	**2**
**Name**	**O’Connell , Malene**
**Affiliation Unit**	**Copenhagen University Hospital, Amager Hvidovre, Gastro**
**Date**	**13-Nov-2024**
**COI**	**No competing interests.**

Thank you for an interesting article. The ALFIE trial is an important addition to the field of evidence-based chronic disease management for persons with decompensated liver cirrhosis, and this study serves as a valuable supplement to the randomized trial. Process evaluations are essential to complement pragmatic randomized trials and complex interventions, as they allow us to learn from negative studies and facilitate replicating positive outcomes.

I do, however, have some questions and recommendations that I believe could strengthen your study:

GENERAL COMMENTS

The study would benefit from applying a process evaluation or implementation framework, which could enhance the study’s impact by offering a structured approach to understanding the intervention's implementation, particularly around feasibility, fidelity, and acceptability. Applying established implementation outcomes and terminology throughout the article could strengthen the study’s rigour and clarity.

Moreover, I’m confused about the inclusion of controls in this study. In my opinion, the result from the controls represents “the before” which might have been more effectively

used as part of the intervention development in the ALFIE trial. I recommend clarifying your rationale for including controls in a qualitative study or considering omitting them.

BACKGROUND, AIM, OBJECTIVES

P. 4. L. 36-37: References 8 and 9 are quite old. The use of qualitative studies and process evaluations to complement randomized trials is becoming more common and is increasingly recognized as essential for understanding how and why randomized interventions work (or do not work) within specific contexts.

I recommend that you change the references and rewrite the section from P. 4 L. 26 to 47 with a focus on the newest literature and evidence in the field.

P. 4 L. 45. In the objective you state that "The study objectives were to analyze how the control and intervention participants perceived the intervention and to examine the views of nurses and doctors who delivered the intervention".

However, since the controls did not receive the intervention, their perceptions would instead reflect the usual care within their setting, and not the intervention itself. This distinction is essential, as it may impact the interpretation of the study's findings.

If you wish to keep the control data in this study, I recommend clarifying the objectives to accurately represent the controls' role, perhaps by reframing the aim to specify that control participants' feedback pertains to usual care rather than the intervention.

METHODS

P. 4-5. The Method section would be improved with the use of subheadings to guide the reader i.e., Design, Participants, Data collection, Data analysis etc.

You state that:

P. 4, L. 58: The data was analyzed on the principles of Systems Theory.

P. 4, L 60: Your previous research on nurse-led clinic in cirrhosis informed the methodology.

P. 5, L. 27: The authors' prior research on nurse-led clinic model in cirrhosis informed the analytical methods used for this study.

P. 5, L. 28: The analysis was performed using Braun & Clarke's six-phase framework for thematic analysis.

It is a lot of different claims on how the data was analyzed. I recommend that you briefly clarify in a data analysis section:

• How do you combine Systems Theory and Thematic analysis?

• How did your previous research inform the methodology?

P. 5, L. 29: A reference for Braun and Clarke's six-phase framework for thematic analysis is missing. Including both a reference and a description of thematic analysis is essential, as it

provides the foundational methodology needed to evaluate your approach to qualitative data analysis.

I would recommend the following to strengthen the clarity and rigour of the analysis section:

• Justification for Thematic Analysis: Please provide a brief explanation of why thematic analysis was chosen for this study.

• Detailed Overview of the Analysis Process: Given that Braun and Clarke's six phases are not a strictly linear process, a more structured and detailed description of your approach to each phase would help readers understand the rigour and flexibility of your analysis process.

P. 5, L. 10: You refer to an initial recruitment plan for the inclusion of 32 participants.

• How did you reach this number?

• Did you consider how to assess when the data collection was completed (I can see a short description in the discussion p. 9, l. 50)?

You further write that you used a purposive sampling strategy. But in the next sentence, you write that you planned to include 32 consecutive participants. The two strategies are opposites.

• Which strategy did you use, and why?

• If you used purposive sampling. How did you select the participants?

RESULTS

A table of participant characteristics is missing. Both on patients and medical staff.

Basic information regarding the interviews is lacking:

• Interview Duration: Please specify the average or range of the interview lengths.

• Post-Intervention interview timing: Clarify how long after the intervention's completion participants were interviewed, as this could influence their perspectives and recall.

Again, I don't think the results from the controls fit in the reporting of the results. If you wish to include control results, these should be reported separately from the intervention participants.

Furthermore, I'm wondering how the results from 14 interviews with participants from the control group are only presented in 5 lines (p. 6 l. 91).

One of the pitfalls described in conducting Thematic Analysis is to use interview questions as the "Themes". In this study, the Subthemes shown in Tables 1 and 2 seem appropriate but the four main themes seem to possibly have been derived from the interview guides and maybe not identified from the data. Some sections seem more like a listing of extracts which could imply a lack of analysis or patterning.

I would consider re-visiting steps 4 and 5 to “identify the ‘essence’ of each theme and constructing a concise, punchy and informative name for each theme” (Clarke, V. & Braun, V. (2013) Teaching thematic analysis: Overcoming challenges and developing strategies for effective learning. The Psychologist, 26(2), 120-123), and possibly redo step 6.

P. 7, L. 52 It is unclear if the section Interlinked Benefits is intended as a theme. The section is though an example of how the data from patients and medical staff could be presented in common themes.

There is a general lack of quotes or extracts from the data in the results section. I recognize that the extracts are presented in the tables, but I believe that the quotes or extracts are an important part of the write-up to help the reader capture the essence of the theme you are presenting.

DISCUSSION

The discussion is relevant and thorough.

The section discussing the controls may need reconsideration based on possible revisions made throughout the study.

ABSTRACT

The abstract of course needs relevant revision after the completion of the major revisions.

**Reviewer**	**3**
**Name**	**O'Brien, Alastair**
**Affiliation**	**UCL, Division of Medicine**
**Date**	**14-Nov-2024**
**COI**	**I have no competing interests.**

I think this study and the ALFIE trial are both very important studies that attempt to address how best to mange patients with decompensated cirrhosis, which represents a substantial unmet need.

I think the paper is well-written and does not require any revision.

I wonder whether the authors might wish to comment a little further on where ALFIE and this qualitative study leaves the field. Do they believe this approach might improve outcomes with refinement. Given their comments on resourcing, would further studies require robust health economic analyses to justify expenditure. Is this model "doomed" to have only a modest impact without adequate support for alcohol use disorder and other mental health problems and perhaps our "medicalised" health care model should be adapted. And playing "Devil's" advocate would our resources be better spent on prevention.

I appreciate that my requests are slightly outside a conventional review. But the paper is good as is and this is such an important area for hepatology and this team have performed the best work, and I would value their thoughts.

VERSION 1 - AUTHOR RESPONSE

Reviewer 1

Methods

1. I understand you wanted to, and did, interview intervention participants and controls, as well as nurses and hep/gastro specialists, but purposive sampling is rather vague and should be elaborated on more. While written with implementation research as a focus, Palinkas et al 2015 (https://www.ncbi.nlm.nih.gov/pmc/articles/PMC4012002/ for example lists a wide variety of purposeful sampling strategies – does yours fall under this, or something else? As written, it seems to be more just a convenience sample of the pre-selected sub-groups. This is fine, but it just needs to be made clearer.

   Thank you for suggesting the article on sampling by Palinkas et al (2015). Although we recruited consecutive patients, as clearly outlined in the protocol published, it would classify as purposive for the following reasons as suggested by Palinkas et al.
   We sought 32 patients to achieve thematic saturation, as a sample of 20-30 is considered adequate in qualitative studies.[1, 2] As patients died during the study, we sought patients who were alive and completed the study. Availability and willingness to participate are important aspects of purposive sampling, as per Palinkas et al.
   The recruitment process went beyond convenience as we sought out interventional and control participants at multiple sites.

2. While it is stated that the interviewers were blinded to the interventional status, would "unblinding" not have occurred during the interviews given participants themselves would have known whether they were in the intervention or control group given it was not possible to be blinded? I think this should be acknowledged and discussed.

   Yes, unblinding was inevitable, as we read through the experiences of participants and for those who interviewed. We will acknowledge this as a study limitation.

3. It is not clear when the interviews took place, were they during or once the intervention had completely finished? Please add this detail
   The interviews were held within 3 weeks of completion of intervention. The detail has been added to the methods.

4. Can you clarify, in table 1, where it does not say "control participants", are these all quotes from the intervention participants. I assume so, but can you make this clearer by labelling in the same way?
   Thanks for pointing this out.
   The table is divided into two sections, 1a and 1b to highlight the themes and quotes from interventional and control participants.

Discussion

5. Your mentioned addiction and mental health in the discussion, were these common among study participants? I cannot access the main paper via my institute, so can you please provide some brief details on this for the participants where you talk about alcohol being the primary aetiology for cirrhosis?

In the main paper that reported on the study outcomes, it was reported that 68% intervention and 69% control participants had alcohol related cirrhosis.[3]
Will add this attachment at the time of online submission.

Reviewer: 2
Dr. Malene O'Connell , Copenhagen University Hospital, Amager Hvidovre
Comments to the Author:
Thank you for an interesting article. The ALFIE trial is an important addition to the field of evidence-based chronic disease management for persons with decompensated liver cirrhosis, and this study serves as a valuable supplement to the randomized trial. Process evaluations are essential to complement pragmatic randomized trials and complex interventions, as they allow us to learn from negative studies and facilitate replicating positive outcomes.

I do, however, have some questions and recommendations that I believe could strengthen your study:

GENERAL COMMENTS
The study would benefit from applying a process evaluation or implementation framework, which could enhance the study's impact by offering a structured approach to understanding the intervention's implementation, particularly around feasibility, fidelity, and acceptability. Applying established implementation outcomes and terminology throughout the article could strengthen the study's rigour and clarity.

Thank you for the important suggestion. We agree with the reviewers that reporting along an implementation framework would be very useful. However, this study was designed with the objective of obtaining the experiences of participants and medical care providers, to find out the strengths and barriers of the intervention. While it did provide answers for a few implementation questions such as feasibility, acceptability, appropriateness, and penetration, the study was not designed with an implementation framework in mind. Adoption and sustainability have to be studied over time and in more centres before the intervention can be implemented. We have added this as a limitation.

Moreover, I'm confused about the inclusion of controls in this study. In my opinion, the result from the controls represents "the before" which might have been more effectively used as part of the intervention development in the ALFIE trial. I recommend clarifying your rationale for including controls in a qualitative study or considering omitting them.

This study is a qualitative assessment of the ALFIE trial which examined a chronic disease management (CDM) intervention in a randomised controlled fashion.[3] 75 participants

received intervention and 71 served as controls. It was essential to include the experiences of recruited participants from both arms, in order to understand the effects of the intervention.

- *If "before" was used, treatment guidelines could have been different*
- *By studying control & intervention participants at same time period, we got more control of the factors that could influenced patient experiences/perspectives*

BACKGROUND, AIM, OBJECTIVES

P. 4. L. 36-37: References 8 and 9 are quite old. The use of qualitative studies and process evaluations to complement randomized trials is becoming more common and is increasingly recognized as essential for understanding how and why randomized interventions work (or do not work) within specific contexts.

I recommend that you change the references and rewrite the section from P. 4 L. 26 to 47 with a focus on the newest literature and evidence in the field.

Noted with thanks. The importance of qualitative methods, interviews of key stakeholders and the case study of SAFER trial wherein qualitative methods were integrated to the main trial are now added. Two new references have been added. And the section amended as highlighted.

P. 4 L. 45. In the objective you state that "The study objectives were to analyse how the control and intervention participants perceived the intervention and to examine the views of nurses and doctors who delivered the intervention".
However, since the controls did not receive the intervention, their perceptions would instead reflect the usual care within their setting, and not the intervention itself. This distinction is essential, as it may impact the interpretation of the study's findings.

Yes, as mentioned the perceptions of controls would reflect the status of usual care. This *mention has been added to the objectives.*

If you wish to keep the control data in this study, I recommend clarifying the objectives to accurately represent the controls' role, perhaps by reframing the aim to specify that control participants' feedback pertains to usual care rather than the intervention.

Yes, it is specified in aims that the control participants' views were sought regarding the standard care offered without the intervention.

METHODS

P. 4-5. The Method section would be improved with the use of subheadings to guide the reader i.e., Design, Participants, Data collection, Data analysis etc.

Yes, subheadings added to improve clarity and presentation

You state that:
P. 4, L. 58: The data was analyzed on the principles of Systems Theory.
P. 4, L 60: Your previous research on nurse-led clinic in cirrhosis informed the methodology.

P. 5, L. 27: The authors' prior research on nurse-led clinic model in cirrhosis informed the analytical methods used for this study.
P. 5, L. 28: The analysis was performed using Braun & Clarke's six-phase framework for thematic analysis.

It is a lot of different claims on how the data was analyzed. I recommend that you briefly clarify in a data analysis section:
• How do you combine Systems Theory and Thematic analysis?

The study is informed by systems theory as described in detail now in the methods section and below.
The study results were analysed on the principles of Systems' Theory as it examined the linear and cyclic interactions between patients and nurses (micro system), between nurses and doctors (interactions between discipline, team delivering model of care (meso system), and their interactions with the health system in hospitals and with GPs in the community (macro), finally where MOC happens, larger system of communication.

The results of thematic analysis – as shown in figure 2, revealed that there were common themes between multiple systems. The themes overlapped across the layers studied.
Systems theory and thematic analysis did not contradict each other.

• How did your previous research inform the methodology?

Previous study by the authors that analysed the qualitative experiences of nurse led model of cirrhosis clinics, [4] provided more perspectives on purposive sampling, broader issues involved in coordinated care, highlighted the layers involved. It also guided the interview questionnaire development and the thematic analysis.

P. 5, L. 29: A reference for Braun and Clarke's six-phase framework for thematic analysis is missing. Including both a reference and a description of thematic analysis is essential, as it provides the foundational methodology needed to evaluate your approach to qualitative data analysis.
I would recommend the following to strengthen the clarity and rigour of the analysis section:

• Justification for Thematic Analysis: Please provide a brief explanation of why thematic analysis was chosen for this study.

The aim of the study was to understand the impact, barriers and strengths of ALFIE intervention. Thematic analysis was the best way to comb through the interview transcripts and identify the various themes or patterns. It was the best way to identify barriers and enablers of the intervention.

• Detailed Overview of the Analysis Process: Given that Braun and Clarke's six phases are not

a strictly linear process, a more structured and detailed description of your approach to each phase would help readers understand the rigour and flexibility of your analysis process.

AP and LW were senior medical students familiar with both patient and nurse interactions and the principles of nurse-led cirrhosis care due to their involvement in similar prior research. They both shared the transcripts and generated initial codes. JR, as a senior author, reviewed all the transcripts and independently coded them (step 1 and 2 of the Braun and Clarke process). The three researchers had a series of online meetings to review all the codes and finalise the themes (2 and 3). The emerging themes were discussed with AW, the principal ALFIE investigator, and SL, an experienced qualitative analyst, before the final themes were decided upon (4 and 5). The research team thus maintained reflexivity during analysis and writing by keeping an electronic record of the discussions. This was followed by the last stage of write up of the manuscript.

P. 5, L. 10: You refer to an initial recruitment plan for the inclusion of 32 participants.
• How did you reach this number?
.
- *There were 4 sites*
- *We wanted a sample size between 20-30, therefore 8 from each site = 32*
- *We assumed that this number would provide us with thematic saturation and provide robust data. There are multiple references for sample size from 12 up to 30 participants for interviews to be able to provide the information required*

• Did you consider how to assess when the data collection was completed (I can see a short description in the discussion p. 9, l. 50)?

You further write that you used a purposive sampling strategy. But in the next sentence, you write that you planned to include 32 consecutive participants. The two strategies are opposites.

• Which strategy did you use, and why?
• If you used purposive sampling. How did you select the participants?

Thank you for suggesting the article on sampling by Palinkas et al . While we recruited consecutive patients as clearly outlined in the protocol published, it would classify as purposive for the following reasons as suggested by Palinkas et al.
We sought 32 patients to achieve thematic saturation as a sample of 20-30 is considered adequate in qualitative studies like ours.[1] As patients died during the study, we sought patients who were alive and completed the study. Availability and willingness to participate are important aspects of purposive sampling, as per Palinkas et al.
The recruitment process went beyond convenience as we sought out interventional and control participants at multiple sites.

RESULTS
A table of participant characteristics is missing. Both on patients and medical staff.
We have added the relevant demographic information collected from the study participants and staff in the results section to adhere to the limit of tables permitted as below.

A purposive number of 29 patients (15 patients and 14 controls, median age 57 years, 66% male) and 12 medical staff (eight nurses and four hepatologists) completed the interviews at the end of the follow-up period. The key clinical characteristics of the participants interviewed were 76% with alcohol-related cirrhosis as primary etiology in 76% and a mean (SD) MELD score of. 16 (6). Nineteen patients were reported to have ascites. All the eight nurses were women and 3 hepatologists were men.

Basic information regarding the interviews is lacking:
• Interview Duration: Please specify the average or range of the interview lengths.

The interview duration varied from 20-40 minutes
• Post-Intervention interview timing: Clarify how long after the intervention's completion participants were interviewed, as this could influence their perspectives and recall.
Participants were interviewed from 1-3 weeks of them completing the study.

Again, I don't think the results from the controls fit in the reporting of the results. If you wish to include control results, these should be reported separately from the intervention participants.

The controls were a part of the important study arm that helped analyse ***the differences due to the intervention****. As suggested the details are separately listed in the table 1b.*

Furthermore, I'm wondering how the results from 14 interviews with participants from the control group are only presented in 5 lines (p. 6 l. 91).

The controls did not have much to talk about the care as it was standard without any intervention. The important themes were included.

One of the pitfalls described in conducting Thematic Analysis is to use interview questions as the "Themes". In this study, the Subthemes shown in Tables 1 and 2 seem appropriate but the four main themes seem to possibly have been derived from the interview guides and maybe not identified from the data. Some sections seem more like a listing of extracts which could imply a lack of analysis or patterning.

I would consider re-visiting steps 4 and 5 to "identify the 'essence' of each theme and constructing a concise, punchy and informative name for each theme" (Clarke, V. & Braun, V. (2013) Teaching thematic analysis: Overcoming challenges and developing strategies for effective learning. The Psychologist, 26(2), 120-123), and possibly redo step 6.

Many thanks for suggesting this.
After discussing with the study team we have concluded that the subthemes actually are the themes that encompass the codes and in turn the quotes.
We have grouped the themes that emerged from controls and intervention participants separately in addition to the staff comments that were already done. We have reworded some themes as per the reviewers' advice to reflect the codes and quotes better.

P. 7, L. 52 It is unclear if the section Interlinked Benefits is intended as a theme. The section is though an example of how the data from patients and medical staff could be presented in common themes.

It is not a theme
It is a summary of common themes.

There is a general lack of quotes or extracts from the data in the results section. I recognize that the extracts are presented in the tables, but I believe that the quotes or extracts are an important part of the write-up to help the reader capture the essence of the theme you are presenting.
We avoided the quotes in the text to adhere to the word limit and to avoid repetition.

DISCUSSION
The discussion is relevant and thorough.

The section discussing the controls may need reconsideration based on possible revisions made throughout the study.
As mentioned before, the inclusion of controls was necessary to explore the lived in experiences of an important aspect of research participants.

ABSTRACT
The abstract of course needs relevant revision after the completion of the major revisions.

The abstract aligns well with the changes in wording of themes.

Reviewer: 3
Dr. Alastair O'Brien, UCL
Comments to the Author:
I think this study and the ALFIE trial are both very important studies that attempt to address how best to mange patients with decompensated cirrhosis, which represents a substantial unmet need.
I think the paper is well-written and does not require any revision.
I wonder whether the authors might wish to comment a little further on where ALFIE and this qualitative study leaves the field. Do they believe this approach might improve outcomes with refinement.
We think the approach has promise despite negative outcome for primary endpoint. We accept that reducing admissions in the setting of sick decompensated patients is going to be difficult, and building a program like ALFIE may in fact attract high value admissions. However, Outcomes may have improved in a different setting (less high quality care in control group, greater resourcing for nursing ie 1.0 not 0.6 fte). Given their comments on resourcing, would further studies require robust health economic analyses to justify expenditure. A robust health economic analysis is to follow.
Is this model "doomed" to have only a modest impact without adequate support for alcohol use disorder and other mental health problems and perhaps our "medicalised" health care model should be adapted. And playing "Devil's" advocate would our resources be better

spent on prevention. Yes-the study has highlighted that inadequate resources for addiction and mental health would be key areas to improve before embarking on a future trial. Its like that other supportive care needs (financial, caregiver, palliative care) may also be relevant to change the trajectory of these patients who are frequently from marginalized settings. Prevention always better than cure but we must also deal with the reality of what is turning up to our hospitals with increasing numbers due to current obesity and alcohol epidemics.

I appreciate that my requests are slightly outside a conventional review. But the paper is good as is and this is such an important area for hepatology and this team have performed the best work, and I would value their thoughts.

References

1. Thomson, S., *Sample Size and Grounded Theory.* JOAAG, 2011. **5**.
2. Marshall, B., et al., *Does Sample Size Matter in Qualitative Research?: A Review of Qualitative Interviews in is Research.* Journal of Computer Information Systems, 2013. **54**: p. 11-22.
3. Wigg, A.J., et al., *A randomized multicenter trial of a chronic disease management intervention for decompensated cirrhosis. The Australian Liver Failure (ALFIE) trial.* Hepatology, 2024.
4. Ramachandran, J., et al., *Nurse Led Clinics; a Novel Model of Care for Compensated Liver Cirrhosis: A Qualitative Analysis.* Gastroenterol Nurs, 2022. **45**(1): p. 29-42.

VERSION 2 - REVIEW

**Reviewer**	**2**
**Name**	**O'Connell , Malene**
**Affiliation**	**Copenhagen University Hospital, Amager Hvidovre, Gastro Unit**
**Date**	**06-Jan-2025**
**COI**	

I appreciate the authors' responses to my comments.

I do however still have some questions and recommendations that I believe could strengthen the work. Especially the analysis and result section still needs major revisions.

METHODS

The method section has been improved immensely with the included subheadings. I do though have a few comments for the different sections:

Design:

- There is a general lack of references for the qualitative descriptive framework and systems theory.

- You state that: The previous research by the authors on a nurse-led clinic model also informed the methodology.[11] (P. 7 L. 57-58). You need to elaborate on how in the article text. You stated this in the reviewer response letter:

Previous study by the authors that analysed the qualitative experiences of nurse-led model of cir-rhosis clinics, [4] provided more perspectives on purposive sampling, broader issues involved in coordinated care, highlighted the layers involved. It also guided the interview questionnaire development and the thematic analysis.

- The following section seems a bit out of place. Should it be moved to the Data collection section?:

P.8 L. 3-6 The interview questions allowed the interviewees (especially the staff) to talk about the systems in which they work, their perspectives on system-level changes that could help the intervention and potential challenges within the service system of care in which they work.

Setting and participants:

- P. 8 L. 7-8: I don't think the sentence fits in this section. Possibly the intervention should have its section with a short description and a reference for the ALFIE trial. Or since the intervention is shortly described and referenced in the background the sentence could be changed to "The settings of the ALFIE trial are described in detail in the initial paper".

- The following section should have a "Recruitment" subheading possibly a joint subheading with data collection.

P. 8 L. 11-15: A purposive sampling strategy was used. The initial recruitment plan was to recruit 32 consecutive participants (8 patients from each major site, with 4 patients from each study arm) over two 5-month periods: October 2020 to February 2021 (Block 1) and October 2021 to February 2022 (Block 2). The interview plan also included interviews with eight nurses and four gastroenterologists/hepatologists, representing four major hospital sites.

- P. 8 L. 11: Purposive sampling should be referenced, possibly with the suggested Palinka reference. You should be aware not to mix up the terms purposive and purposeful sampling since these can have different meanings depending on the reference. Palinka uses the term Purposeful. I suggest you briefly elaborate in the text what you mean by purposive or purposeful.

Data collection:

P 8. L. 19: Since you state further in your study that it was not possible to blind the interviewers, I think it would be relevant to add "sought to be blinded" or possibly omit the sentence but keep the limitation further on in the study.

P. 8 L. 47-51: This section should be moved to an ethical consideration section.

RESULTS

Participant characteristics:

- Since you are meant to compare the control and intervention groups it could be useful to show how the groups compare. It could affect the results if the groups differentiate in gender, MELD score or etiology.

- You have included the number of participants presenting with ascites. Since the ALFIE trial showed a reduction in HE admissions it could be relevant to present the number of included participants presenting with HE.

- The years of work experience and years of experience in the hepatology field would be relevant to add for the staff characteristics.

I still have some concerns about the inclusion of the controls in the study.

You state that:

It was essential to include the experiences of recruited participants from both arms, to compare and understand the effects of the intervention.

and

We got more control of the factors that could influenced patient experiences/perspectives.

The separation of the objectives and results between intervention- and control participants has improved the understanding, but I don't believe you answered your objectives and used the results from the controls to compare and understand the effect of the intervention.

For now, the results from the control group interviews are listed in the result section. Since the comparison between groups is part of your objective it should be addressed in both the results, discussion, conclusion and possibly figures.

A suggestion could be to start the result section with the results from the controls and show in the following section and/or in a figure or table how the interventions have solved some of the issues described.

I appreciate that you have revisited the analysis. I do though still believe that the analysis needs some work:

- Some of the themes lack description for example "Paperwork", and "Education". Remember that the themes in thematic analysis should tell a story.

- Furthermore, some themes seem to cover a more overarching theme. For example, "Holistic care", "Personalised care", "Continuous care", and possibly "Care navigation" could be included under one overarching theme.

I would again consider re-visiting steps 4 and 5 to "identify the 'essence' of each theme and constructing a concise, punchy and informative name for each theme" (Clarke, V. & Braun, V. (2013) Teaching thematic analysis: Overcoming challenges and developing strategies for effective learning. The Psychologist, 26(2), 120-123), and redo step 6.

ABSTRACT

The results section needs a better structure. The sentence concerning the controls se

---

VERSION 2 - AUTHOR RESPONSE

Please find our responses to each of the questions point by point.

METHODS
The method section has been improved immensely with the included subheadings. I do though have a few comments for the different sections:

Design:
- There is a general lack of references for the qualitative descriptive framework and systems theory.

Thanks for this suggestion.

We have provided references and further details in the methodology section

- You state that: The previous research by the authors on a nurse-led clinic model also informed the methodology.[11] (P. 7 L. 57-58). You need to elaborate on how in the article text. You stated this in the reviewer response letter:

Amended as suggested
Previous study by the authors that analysed the qualitative experiences of nurse-led model of cirrhosis clinics[4] provided more perspectives on purposive sampling, broader issues involved in coordinated care and highlighted the layers involved. It also guided the interview questionnaire development and the thematic analysis.

- The following section seems a bit out of place. Should it be moved to the Data collection section?:
P.8 L. 3-6 The interview questions allowed the interviewees (especially the staff) to talk about the systems in which they work, their perspectives on system-level changes that could help the intervention and potential challenges within the service system of care in which they work.

Amended as suggested

Setting and participants:
- P. 8 L. 7-8: I don't think the sentence fits in this section. Possibly the intervention should have its section with a short description and a reference for the ALFIE trial. Or since the intervention is shortly described and referenced in the background the sentence could be changed to "The settings of the ALFIE trial are described in detail in the initial paper".

- The following section should have a "Recruitment" subheading possibly a joint subheading with data collection.

Amended as suggested

P. 8 L. 11-15: A purposive sampling strategy was used. The initial recruitment plan was to recruit 32

consecutive participants (8 patients from each major site, with 4 patients from each study arm) over two 5-month periods: October 2020 to February 2021 (Block 1) and October 2021 to February 2022 (Block 2). The interview plan also included interviews with eight nurses and four gastroenterologists/hepatologists, representing four major hospital sites.

- P. 8 L. 11: Purposive sampling should be referenced, possibly with the suggested Palinka reference. You should be aware not to mix up the terms purposive and purposeful sampling since these can have different meanings depending on the reference. Palinka uses the term Purposeful. I suggest you briefly elaborate in the text what you mean by purposive or purposeful.

Added as suggested

Data collection:
P 8. L. 19: Since you state further in your study that it was not possible to blind the interviewers, I think it would be relevant to add "sought to be blinded" or possibly omit the sentence but keep the limitation further on in the study.

The sentence was omitted as suggested

P. 8 L. 47-51: This section should be moved to an ethical consideration section.

Amended as suggested

RESULTS
Participant characteristics:
- Since you are meant to compare the control and intervention groups it could be useful to show how the groups compare. It could affect the results if the groups differentiate in gender, MELD score or etiology.

- You have included the number of participants presenting with ascites. Since the ALFIE trial showed a reduction in HE admissions it could be relevant to present the number of included participants presenting with HE.

Since the ALFIE intervention was offered in a randomised manner, the control and intervention participants were matched in age, gender and MELD scores with no significant differences between them. This has been demonstrated in the original publication.

We have provided the details of clinical presentation requested for the participants.

- The years of work experience and years of experience in the hepatology field would be relevant to add for the staff characteristics.

The hepatologists had up to 10 years of experience and the nurses had upto 5 years experience as chronic hepatitis nurses and many years' experience in nursing . The exact years of experience was

not within the scope of our data collection. It is important to note that none of the nurses had any prior experience in chronic liver failure or chronic disease management management. They received training before of trial and ongoing training and support from the trial team during the trial. We have included this information now in the manuscript.

I still have some concerns about the inclusion of the controls in the study.

You state that:
It was essential to include the experiences of recruited participants from both arms, to compare and understand the effects of the intervention.

and

We got more control of the factors that could influenced patient experiences/perspective--**Happy to omit this sentence**

We studied the effect of the RCT and it involved both controls and intervention patients and hence we designed this study to include both control and interventional participants. This gave us a unique and valuable opportunity to compare patient experience is our usual care versus intervention model.

The separation of the objectives and results between intervention- and control participants has improved the understanding, but I don't believe you answered your objectives and used the results from the controls to compare and understand the effect of the intervention.

For now, the results from the control group interviews are listed in the result section. Since the comparison between groups is part of your objective it should be addressed in both the results, discussion, conclusion and possibly figures.

A suggestion could be to start the result section with the results from the controls and show in the following section and/or in a figure or table how the interventions have solved some of the issues described.

Many thanks for this important suggestion. As per your advice, we have highlighted the differences between controls and intervention participants in discussion and conclusion. We have described the control participants' findings after the results of intervention participants to draw attention to the interventional effects.

I appreciate that you have revisited the analysis. I do though still believe that the analysis needs some work:

- Some of the themes lack description for example "Paperwork", and "Education". Remember that the themes in thematic analysis should tell a story.

- Furthermore, some themes seem to cover a more overarching theme. For example, "Holistic care", "Personalised care", "Continuous care", and possibly "Care navigation" could be included under one overarching theme.

I would again consider re-visiting steps 4 and 5 to "identify the 'essence' of each theme and constructing a concise, punchy and informative name for each theme" (Clarke, V. & Braun, V. (2013) Teaching thematic analysis: Overcoming challenges and developing strategies for effective learning. The Psychologist, 26(2), 120-123), and redo step 6.

Many thanks for this suggestion. We have added punchy informative names for each category in the results section. This is because, the structure of these sections is chiefly to summarise across the themes rather than provide descriptors of each in more detail, and to refer the reader to the Tables for detail. The study team felt that our themes appropriately represented the perspectives of intervention participants and staff. We felt that the themes continuous care, personalised care and holistic care and care navigation are distinct elements of care. They are well described by the mentioned quotes and were important enablers of improved care.

ABSTRACT
The results section needs a better structure. The sentence concerning the controls se

The abstract section has been structured as per the journal's recommendation including all the relevant findings. We have reviewed the abstract and modified its expression to improve structure and clarity.

VERSION 3 - REVIEW

**Reviewer**	**2**
**Name**	**O'Connell , Malene**
**Affiliation**	**Copenhagen University Hospital, Amager Hvidovre, Gastro Unit**
**Date**	**22-Jan-2025**
**COI**	

Thank you for all your changes. I truly believe the article has improved immensely since the first submission. I hope you agree

I only have a few suggestions for (very) minor changes.

Congratulations on a great study. The ALFIE trial and this qualitative study are significant contributions to the field of outpatient treatment and care of patients with decompensated liver cirrhosis.

Comments:

Throughout the text, you need to check for extra full stops, commas, parentheses, spaces, etc.

P. 7 l. 18-24 There is no references for this section. You could include a reference on Complex interventions, and/or implementation publications showing the importance of process evaluations and the inclusion of qualitative research.

p. 8 l. 29-32 I suggest you either create to descriptions, one for the staff and one for patients, or you change the sentence to be broader – like the example below:

The interview questions were designed to allowed the interviewees (especially, the staff) to talk about the systems in which they work and their experiences with the intervention, perspectives on system-level changes that could help improve the intervention, and also potential challenges within the service system of care in which they worked.

p. 9 l. 14. I suggest deleting the section concerning the themes, since this is covered in the sections below. See the suggested changes below:

The baseline characteristics of the study participants are presented in Table 1. Baseline characteristics of study participants. 29 patients (15 patients and 14 controls, median age 57 years, 66% male) and 12 medical staff (eight nurses and four hepatologists) completed the interviews at the end of the follow-up period. 76% of the interviewed participants had alcohol-related cirrhosis with a mean (SD) MELD score of 16 (6). Fifteen participants (8 control and 7 intervention presented with ascites and three (two control and one intervention) had hepatic encephalopathy at initial admission. All the eight nurses were women and the three hepatologists were men. The interviews lasted from 20 to 40 minutes and were conducted within 3 weeks of study completion.

Themes that emerged from these interviews reflected a range of enablers and barriers to the effective delivery, positive experience of the intervention, as perceived by participants and staff and needs for better care by the controls. Table 1. Baseline characteristics of study participants

p. 9 l. 19-20, p. 14 l. 4-5

I suggest dividing the headlines in two different sections/levels (see below), and possibly adding the overarching themes to the tables 3 and 4. You could consider another word than "value/valuing" in one of the themes, just to create variety.

Themes from interventional participants' Interviews

The value of personalised, holistic and continuous care

Themes from Medical Staff's Interviews

Valuing holistic care with complex and challenging systems

P.10 – 18 Results and analysis:

I really appreciate the thoughts about, and changes made in the analysis, result, and discussion section.
